# A review of large biochemistry analysers

**DOI:** 10.1155/S1463924688000112

**Published:** 1988

**Authors:** John Fyffe, P. Daga, M. J. Roddis

**Affiliations:** 1 Clinical Biochemistry Queen Elizabeth II Hospital Howlands Welwyn Garden AL7 4HQ UK; 2 Chemical Pathology Ashford Hospital Ashford Middlesex UK; 3 Chemical Pathology Edgware General Hospital Edgware Middlesex HA8 0AD UK


					Journal of Automatic Chemistry, Vol. 10, No. (January-March 1988), pp. 43-61
A review of large biochemistry analysers
John Fyffe,
Clinical Biochemistry, Queen Elizabeth II Hospital, Howlands, Welwyn Garden
City, AL7 4HQ UK
P. Daga
Chemical Pathology, Ashford Hospital, Ashford, Middlesex, UK
and M.J. Roddis
Chemical Pathology, Edgware General Hospital, Edgware, Middlesex
HA80AD, UK
Introduction
In recent years the numbers of large biochemistry
analysers on the UK market has significantly increased
and it was felt that a review of their specification in a
comparative form may help prospective purchasing
laboratories select from the large number of analysers
available those that would be relevant for their purpose.
Questionnaire
To this end a questionnaire was sent out inJanuary 1987
to 80 companies in the UK known to be involved in
supplying biochemistry laboratories, requesting that a
copy of the questionnaire be completed for each analyser
they support on the UK market. The analyser was
defined as being able to produce more than 100 test
results for any of a group of the 23 most commonly
performed biochemistry tests. Twenty-one companies
replied positively, providing information for 69 analysers,
which is presented in tables 1-16.
Results
Each table represents the manufacturer's answer to
questions on one or more subjects listed at the head of
each column. All information presented here has been
checked with the manufacturer, however, it should be
stressed that the information has come from manufactur-
ers and its validity has not been checked by the authors.
This exercise was carried out earlier, Fyffe in 1985 and
an attempt has been made to correct as many anomalities
as possible. It is important that the information presented
be used as a guide to specifications of analysers and
prospective purchasers should check all these details with
the appropriate manufacturer before proceeding to pur-
chase.
Questionnaire
1.1 Manufacturer and model.
1.2 When was this model first available in UK?
1.3 Which tests have available recommended methodologies?
1.4 How long do you expect this instrument to be available in the UK?
2.1 Which samples are acceptable?
2.2 What is the sample tray/carriage capacity?
2.3 What sample volumes are required to perform the stated groups ofanalytes? U&E Na, K, C1, Bic, Urea,
Creat. LFT Bili, T Prot, Alb, ALP, +3 other Enzymes. BONE Ca, PO4, Alb, ALP. CARD 3
Enzymes. MISC Cholesterol, Triglyceride, Urate. GLU Glucose.
3.1 How many samples ofeach analyte group could be handled per hour, including calibration and QC? U&E
Na, K, CI, Bic, Urea, Creat. LFT Bili, T Prot, Alb, ALP, +3 other Enzymes. BONE Ca, PO4, Alb,
ALP. CARD 3 Enzymes. MISC Cholesterol, Triglyceride, Urate. GLU Glucose.
3.2 Are tests performed in batches?
3.3 What is the maximum batch size? (Where applicable.)
3.4 Does the instrument analyse samples selectively, in profiles or both?
3.5 What is the maximum number of samples/tests which can be analysed without operator intervention?
3.6 Has the instrument been the subject of a DHSS or other independent evaluation? If so, please quote.
3.7 What is the claimed within and between batch imprecision for each analyte.
4.1 What is the recommended frequency of calibration?
4.2 (a) Do you recommend aqueous or serum based calibrant?
4.2 (b) Can the instrument be calibrated by the operator using his existing assayed material?
4.2 (c) How many calibrators are required? Please state max/min.
43
j. Fyffe et al. Review of large biochemistry analysers
5.1 Can the instrument be safely used for handling high risk samples? (For example Hepatitis B, AIDS.)
NB. In a normal clinical laboratory environment and using normal laboratory practices.
5.2 Is there a recommended method of decontamination in normal use and before planned maintenance?
5.3 Does the instrument comply with the ESCHLE code for electrical safety? (Ref: Health Equipment Information,
No. 140, April 1985).
5.4 Please state any BSI standard with which the instrument complies.
6.1 Can the routine work be interrupted for stat samples?
6.2 How long would it take to produce a U&E and Glucose result from (a) Start up (b) Standby? U&E Na,
K, C1, Bic, Urea, Creat, and glucose.
7.1 What services are required for installation?
7.2 Is the instrument floor or bench standing?
7.3 How much space is required including service access? (In m.)
7.4 What is the weight? (In kg.)
7.5 Are operator training courses available? If so (a) how long, (b) how many staff, (c) what is the cost?
7.6 How many instruments are installed in the UK?
8.1 (a) What is the downtime per year for planned maintenance, not including daily maintenance?
(b) How long does daily maintenance take (for example of weekly divided by 5 etc.)?
8:2 Which types of service contract are available and at what cost?
8.3 Does the cost of service contract depend on the use of a specific manufacturer's reagents, spares or
consumables?
8.4 How long is the warranty?
8.5 Does the warranty depend on the use of a specific manufacturer's reagents, spares or consumables?
8.6 How long do you expect to support maintenance of the instrument?
9.1 (a) In its standard form without any optional data manager, can the instrument log/monitor/provide
statistics for quality control results?
(b) As above but with a data manager.
9.2 (a) In its standard form can the instrument hold patient identification and results?
(b) As above but with a data manager.
9.3 (a) In its standard form what is the storage capacity for QC/PID/RESULTS?
(b) As above but with a data manager.
9.4 (a) Is the standard instrument fitted with an RS 232/232C compatible ONE way port?
(b) As above but with a TWO way port.
(c) If not, please quote price for (a) and (b).
9.5 (a) In its standard form is the report format fixed or user definable for printing results/PID on various sizes
of paper?
(b) As above but with data manager.
9.6 (a) In its standard form is it possible to add results produced on other equipment to the report? (i) For on
board chemistries (ii) For other chemistries produced on other instruments?
(b) As above but with a data manager.
9.7 (a) In its standard form can the instrument produce statistics required for the Korner report including
listings for (i) Consultant, (ii) Locality, for example ward, and (iii) Clinical Specialty?
(b) As above but with a data manager.
9.8 (a) Is help available to link the analyser to an existing or future laboratory computer system?
(b) If so, what is the approx cost of (i) hardware (ii) software?
10.1 What is the basic price of the instrument? (In pounds sterling.)
10.2 (a) Cost of the ISE unit?
(b) Cost of data manager?
NB. State if included in 10.1.
10.3 What is the expected recommended reagent cost to perform each group of analytes (see below) on 100
samples?
10.4 If 100 samples were analysed for each of the analyte groups (see below), what would be the cost of the
consumables used in the analytical process?
10.5 (a) What is the volume of calibrant required to calibrate all on board chemistries once?
(b) What is the cost per ml. of the recommended calibrant?
(c) What is the reconstituted volume and stability of the recommended calibrant?
U&E Na, K, C1, Bic, Urea, Creat. LFT Bili, T Prot, Alb, ALP, +3 other Enzymes. BONE Ca, PO4,
Alb, ALP. CARD 3 Enzymes. MIS Cholesterol, Triglyceride, Urate. GLU Glucose. NB. for ALL,
not all tests may be available at any one time.
44
J. Fyffe et al. Review of large biochemistry analysers
NO 1.1
ABBOTT VP SUPER
AHERICAN NITOR PARALLEL
3 AHERICAN NONITOR PERSPECTIVE
BAKER ENCORE II
5 BAKER SPIRIT
6 BCL HITACHI 704
BCL HITACHI 705
8 BCL HITACHI 737
9 BDH EPPENDORF EASY
10 BDH EPPENDORF EP08
II BDH EPPENDORF ERIS
12 BDH EPPENDORF EXSEL
13 BDH EPPENDORF EXTRA
14 BDH OLYHPU8 AU5021
15 BDH OLYHPU8 AUSI2I
16 BDH OLYHPU8 AUS031
17 BDH OLYHPUS AUSI31
18 BOH OLYHPUS AUS041
19 BDH OLYHPUS AUSI41
20 BDH OLYHPUS AUS061
21 BDH OLYHPUB AUS081
22 BECKHAN SYNCHRON AS4
23 BECKHAN SYNCHRON AS8
24 BECKNAN SYNCHRON A8 ENZYHE
25 BECKHAN SYNCHRON AB LIPID
26 BECKHAN SYNCHRON AS H]NI IDEAL
27 BECKHAN SYNCHRON AS IDEAL
28 BECKHAN 8YNCHRON CX3
29 DECKHAN SYNCHRON CX4
30 BECKHAN SYNCHRON CX5
31 DECKHAN SYNCHRON CX7
32 BIO AUTOHATED ASCA
33 CHEHLAD CHEHILYSER
34 CHEIILAD 8YSTEH4
35 CLIN[CON PRISHA lI
36 CIBA CORNIN6 580 ALLIANCE
37 CIDA CORNIN8 570
38 CIBA CORNIN6 EXPRESS
39 COULTER CPA
40 COULTER DACOS
41 DUPONT DIHENBION 380
42 DUPONT DIHEN$ION 760
43 6REINER 6-400
44 6REINER 6-450
45 6REINER TANDEH
46 6REZNER TANDEH 3
47 IL 6ENESZ8 21
48 ]L NONARCH
49 [L HONARCH PLUS
50 KODAK EKTACHEH DT
51 KODAK EKTACHEH 700
52 KONE PRO6RESS
53 KONE SPECIFIC
54 NOVA BIOHED 1.1
55 NOVA BIOHED 4+4
56 NOVA BIOHED 5+5
57 ROCHE CODA8 FARA
58 ROCHE CODAS HIRA
59 TECHNICON ASSIST
60 TECHNZCON CHEH
61 TECHNICON RA-IO00
62 TECHNICON RA-2X
63 TECHNICOR RA-500
64 TECHN]CON RA-XT
65 TECHN]CON II
66 TECHN]CON ORA 2000
67 ULTROLAB ENI 6EH PROFILER
tdi ULTRoLAB ULTROCHEH
69 VITAL SCIENTIFIC VITALAB 200
1.2 1.3
1979/83 19/21+20+E HAX NO Na/K
HID 1982 22/22/3
SEP 1985 22/22+8
JAN 1985 19/22/74+E
1987 21/22
1986 22/22+21+E
1961 21/21+14+E
1985 21/21+16/E
JAN 1996 21/22+7+E
JAN 1985 20/22/19/E NO Na/K
HAY 1984 22/22+14 HAX 23
JUN 1995 22/22+19/E HAX 24
JUN 1985 22122+14 HAX 43
HAY 1967 22/22+19+E HAX 27
HAY 1987 22/22+19+E HAX, 19
HAY 1987 22/22+19+E HAX 27
BAY 1967 22/22+19/E HAX 14
HAY 1997 22/22+19+E HAX 35
HAY 1967 22/22+19/ HAX 19
HAY 1967 22/22+19+E HAX 26
BAY 1907 22/22+19+E HAX 34
1980 13/22 HAX 5
1981 13/22 HAX
1982 22/22 HAX 13
1963 22/22 HAl 11
1983 22/22 flAX 17
1983 22/22 HAX 23
EARLY 87 22/22 HAX 8
EARLY 89 22/22 HAX 24
EARLY 88 22/22 HAX 26
LATE 86 22/22 HAX 32
1997 19/22+5l+E
8EP 1992 15/22 HAX
1983 15/22 HAX
1996 22/22+7+E HAX 46
HID 1987 22122+2
LATE 87 21/22+3
LATEI967 19/22
HAR 1987 19/22+4 HAX 8
AU6 1993 22/22+6 HAX 30
HIO 1906 19/22+10 HAX 37
HID 1986 19/22+18 HAX 37
1993 22/226 HAX 30
1986 22/22+9 HAX 30
1996 22/22+39 HAX 60
HAX 30
HAX 64
NO Na/K
HAX 20 NO HBDH
NO Na/K
1986 22/22+39 HAX 90
FED 1985 22122+5/E HAX 21
SEP 1985 22/22+20+E HAX 23
1995 22/22/20+E HAX 23
1905 10/21+4
1997 20/21+10
HAR 1984 22/22+30/E HAX 27
SEP 1986 22/22+30
1977 Ha/K ONLY
1991 Na/K/CI/D|c ONLY
1902 Na/K/CI ONLY
1984 22/22+10/E
1984 22/22/10+E
1997 17122 HAX 12 NO Na/K
JUN 1965 22/22/3 HAX 35
1963 22/22/60/E HAX 15
1964 22/22/80/E IIAX 27
1985 20/22/60/E HAX 12 NO Na/K
1996 22/22/00/E HAX 26
1991 21/22/2 HAX 23
1993 22/22/00/E HAX 35
JAN 1967 20/22 NO Na/K
JUL 1907 NIDA
JUN 1967 19/22/3/E HAX 40
1,4
1990+
7-10Y
7-10Y
IOY
INDEFF
7Y
RECONDIT
5Y
)IOY
>IOY
>IOY
)IOY
)IOY
>IOY
)IOY
>IOY
>IOY
>IOY
>tOY
>IOY
)IOY
6Y
6Y
Y
Y
Y
Y
IOY
IOY
IOY
tOY
INDEFF
>tOY
>IOY
5Y
5Y
5Y
INOEFF
FUR NOT
UPTOIOY
UPTOIOY
>SY
>IOY
>IOY
7-10Y
7-lOY
7-10Y
INDEFF
[NDEFF
5Y
5Y
5Y
5Y
7Y
>7Y
>7Y
>7Y
>7Y
>7Y
>7Y
)7Y
>7Y
)IOY
N/DA
)tOY
45
j. Fyffe et al. Review o'f large biochemistry analysers
NO INST 2,1 2,2 2.3 UIE 2,3 LFT 2.3 BONE 2.3 CARS) 2,3 HISC 2.3 OLU
ABBOTT VPS PSU 32 NO UIE 44 24
PARALLEL PSU 150 75 135 45
PERSPECTIVE PSU 40 98 84 30
ENCORE II PSU 96 NO UIE 59 19
SPIRIT PSU 40 161 110 27
HITACHI 704 PSUNB 50
HITACHI 705 PSUNB 48 119 58 23
HITACHI 737 PSIAIB 60 37 44
9 EASY PSU 120
10 EPOS PSU 200 NO UIE SS 22
11 ERIS PSU 100 84 22
12 EXSEL PSU 300 84 22
13 EXTRA PSU 200 84 22
14 AUS021 PSU 300 $1 22
15 AUSI21 PSU 300 81 22
16 AUS031 PSU 300 81 22
17 AUSI31 PSU 300 81 22
18 AU5041 PSU 300 81
19 AUSI41 PSU 300
20 AUS061 PSU 300 81
21 AUS081 PSU 300 81
22 AS4 PSU 38
23 AS8 PSU 38 llO
24 AS ENZYNE PSU 38 110
25 AS LIPII) PSU 38 110
26 NINI II)EAL PSU 38 110
27 AS II)EAL PSU 38 110
2B CX3 PSU BO 112
29 CI4 PSU 80 N/I)A
30 CX5 PSU 80 N/DA
31 CX7 PSU 80 N/I)A
32 ASCA PBU 48 20
33 CHEN]LYSER PSU 80
34 SYSTEH4 PSU 300
3 PRI$A II PSU 200 300
18 18
128 II
59
61
40
30
29
230 N/DA 120
45
91 60
98 45
91 60
73 45
73 45
73 45
73 45
73 22 45
73 22 45
73 22 45
73 22 45
144 48 85
144 48 05
144 46 85
5
6 2
15
27
25 15
15
12 6
N/DA 5
lO 5
17 5
10
17
I0
lO 5
10
lO 5
10 5
10 5
10 5
10 5
10
10
10
30 10
30 10
30 I0
N/I)A N/OA N/I)A N/I)A N/I)A
N/DA N/DA Nli)A N/DA N/DA
N/I)A N/DA N/I)A N/DA N/I)A
lO00 200 300 200
36 CORNIN6 580 PSU
37 CORNIN6 570 PSU
36 CORNIN6 EXPRESS PSU
39 CPA PSU 32 NO U/E 97
40 DACOS 2 PSU 90 150
41 DIHENSIOH 360 PSU 60 126
42 I)IHENOION 760 PSU 120 129
43 6REINER 6-400 PSU 30 145
44 6REIHER 6-450 PSU 30 145
45 TANOEH 2 PSU 60 145
46 TANDEH 3 PSU 90 145
47 6ENEBIS 21 PSU 50 82
49 HONARCH PSU 39 46
49 HONARCH PLUS 2 PSU 76 46
50 EKTACHEH DT PS N/AP 70
$! EKTACHEB 700 PSU 40 60
52 PRO6REBS PBU 84 145
53 SPECIFIC PSU 94 145
54 NOVA DIOHED I/1 PSUHD 40
55 NOVA DIOHED 4+4 PSUND 40
56 NOVA DIOHED 5*5 PSUHD 40 4}
57 CODAS FARA PSU 150 105
58 CODAS NIRA PSU 90 105
90 135 235 145 185 130 117
90 N/OA N/OA N/DA NIDA H/DA Nli)A
40 N/DA N/DA N/DA N/DA N/DA N/DA
59 ASSIST
60 CHEH
61 RA-IO00
62 RA-2X
64 RA-XT
65 SNAC II
66 SRA 2OO0
23 49 16 4
46 19 14 10 3.
156 17 66 23 3
156 17 68 23 3
125 35 60 30 5
125 35 60 30 5
125 35 60 30 5
125 35 60 30 5
132 30 45 19 5
70 20 20 11
70 20 20 II 3
60NO ALD N/A 30 30 10
70 41 33 30 lO
31 8 20 10 2
31 6 20 10 2
78 17 26 15
109 17 43 15
50 35 5
50 35 5
50 35 5
50 35 5
50 35 5
PGU 16 NO UlE 90 30
PSU 268 13
PSU 30 35 90 30
Pgu 60 35 90 30
PSU 30 NO UlE 90 30
PSIJ 30 35 90 30
PS 144 450 450 450 450 450 450
PSU 174 35 90 30 50 35 5
67 SEH PROFILER 8PU 45 NO UlE PRO6 PRO6 PRO6 PRO6 PRO6
66 ULTIKiCIH Nli)A 100 NO UIE 100 35 60 20 ,
69 VITALAD 200 PSU 12 NIDA N/DA NIDA NIDA NIDA NIDA
2.3 ALL
104 NO U/E
394
281
126 NO U/E
386
152
222
109
N/DA
170 NO UIE
279
254
274
239
239
239
239
239
239
239
427
N/DA
N/DA
N/I)A
230 NO U/E
2000
325
NIDA
N/DA
180 NO U/E
279
325
325
325
325
27!
157
284
214
214
243
299
240 NO U/E
240
240
240 NO UIE
240
450
240
PRO6
Nli)A
46
j. Fyffe et al. Review of large biochemistry analysers
NO INST :3.! U/E :3.1 LFT
ABBOTT VPS NO U/E
PARALLEL 240
:3 PERSPECTIVE 200
ENCORE II NO UIE
5 SPIRIT 40
6 HITACHI 704 60
HITACHI 705 60
8 HITACHI 737 75
EASY 15
10 EPOS NO UIE
11 ERIS 100
12 EISEL 100
1:3 EXTRA 200
14 AU5021 100
15 AU5121 150
16 AU5031 150
17 AUSI31 300
18 AU5041 150
19 AU5141 :300
20 AU506! :300
21 AU5081 300
22 AS4 65
23 AS6 65
24 AS ENZYHE 65
25 AS LIPID 65
26 HINI IDEAL 65
27 AS IDEAL 65
29 CX:3 75
29 CX4 NO U/E
:30 CX5 75
:31 CX7 75
:32 ASCA NO U/E
:33 CHEH|LYBER 120
:34 SYSTEH4 90
35 PRISHA |I :300
36 CORN]NO 560 100
37 CORNIN6 570 70
39 CORHIN6 EXPRESS NO U/E
39 CPA NO UIE
40 DACO 45
41 DIHENBION :380 46
42 DIHENOION 760 95
43 6REINER 6-400 42
44 6REINER 6-450 57
45 TANOEH 2 114
46 TANDEH 3 170
47 6ENESi8 21 33
46 HONARCH 60
49 HONARCH PLUS 2 120
50 EKTACHEH OT
51 EKTACHEN 700 90
52 PRO61IES6 40
5:3 SPECIFIC NO U/E
54 NOVA BION[D l/l
55 NOVA BIOD 4/4
NOVA BIOHED 5/5
57 COBB FARA 36
56 CODAS HIRA 18
5g ASSIST Hi} UIE
60 CHEH 240
61 RA-IO00 60
62 RA-2X 120
63 RA-500 NO U/E
64 RA-XT 60
65 911AC II 150
66 9RA 2O00 150
67 H PROFILER NO U/E
66 ULTRoLAD NO U/E
69 VITALAD 200 NO UIE
BONE 3,1 CARD :3.1 HISC 3,1 6LU
44 64 93 77 465
240 240 240 240 240
100 200 300 :330 500
57 100 13:3 100 N/DA
:30 60 40 60 NIDA
:30 45 90 60 180
30 45 90 60 180
150 :300 :300 300 :300
8 15 20 20 50
39 75 100 66 :300
59 100 1:3:3 13:3 400
96 175 233 201 700
118 200 266 266 800
tO0 100 100 100 tO0
150 150 150 150 150
150 150 150 150 150
300 300 300 :300 :300
150 150 150 150 150
:300 300 300 :300 300
:300 :300 300 :300 :300
300 300 :300 :300 :300
N/DR N/DR N/DA N/OR 65
NIDA N/DA N/DA NIDA 65
50 50 50 NIDA 65
N/DR N/DA N/DA 50 65
50 50 50 50 65
50 50 50 50 65
NIDA N/OR N/DA N/DA 75
N/DA H/DA N/DA H/DA N/DA
N/DA N/DA N/DA N/DA N/DA
N/DA H/DR N/DA N/DA H/DA
:30 55 7:3 55 N/DA
NIDA 120 N/DA N/DA 80
N/OA N/DA N/DA N/OA 60
:300 :300 :300 :300 :300
100 tO0 100 100 100
60 105 100 100 300
26 45 60 60 180
22 :33 60 60 220
64 112 150 150 450
25 :35 40 45 170
50 70 60 90 340
37 75 100 100 300
50 100 133 133 400
100 200 266 266 600
150 :300 400 400 1200
25 45 60 60 200
55 100 140 135 250
110 200 280 270 500
6 NIDA 6 21 65
71 90 120 120 :360
26 45 60 60 N/DA
20 :3:3 4:3 4:3 N/DA
28 56 70 67 N/DA
19 22 4:3 44 N/DA
10 15 20 20 60
10:3 180 240 240 720
34 60 80 80 240
69 120 160 160 460
34 60 60 80 240
34 60 60 80 240
150 150 150 150 150
150 150 150 150 150
NIDA N/DA N/DA NIDA NIDA
20 35 40 50
10 18 24 24 70
47
jo Fyffe et al. Review of large biochemistry analysers
NO INST
ABBOTT VPS
PARALLEL
3 PERSPECTIVE
ENCORE II
5 SPIRIT
6 HITACHI 704
HITACHI 705
6 HITACHI 737
? EASY
10 EPOS
11 ERIS
12 EXSEL
1 EXTRA
14 AU021
1 121
16 AUS031
17
19 ANS041
20 AUSOI
21 5081
22 AS4
2)
24 AS ENZYHE
25 AS LIPID
2 nlHl IOEAL
27 AS IOEAL
28 CX3
29 CX4
0 CX5
1 CX7
32 ACA
3 CHEILYER
34 YTE4
36 CORNINO 580
37 CORNIN6 570
38 CNINO EXPRESS
3 CPA
40 D
41 DINSI
42 DINSION
4 6REINER 6-400
44 EIR 6-450
45 TDEH 2
4 TANDEN 3
47 81 21
47 NONARCH PL 2
5 EKTACHER OT
51 EKTKHEN 700
52 PRE9
5 ECIFIC
54 VA BIED 11
A BID 4,4
5 NA 8ID 5+5
7 COS FA
59 ASSIET
0 CHEN
1 RA-IOO0
2 RA-2X
RA-500
4 RA-XT
SAC II
SRA 20
7 N PRILER
8 ULTRAS
q VITKA8 2
:3.2 :3.3
YES 28
NO
CAN DE 40
YES 30
YES 40
NO N/APP
NO N/APP
NO N/APP
NO
YES
NO
200
CAN BE 200
NO
NO
NO
NO
NO
NO
NO
NO
NO
NO
NO
NO
NO
NO
NO
NO
NO
NO
NO
CAN DE 36 lO00
YES 460
YES 300
CAN BE
NO N/A
NO N/A
NO N/A
YES 64
NO
CAN
CAN
CAN
CAN
CAN
NO
CAN DE 36
CAN DE 38
NO
NO
CAN
YES
YES
YES
YES
YES
CAN
NO
HO
YES
YES
BOTH
CAHDE 100
DE 55
DE 55
DE HO LIHIT
BE NO LIHIT
BE NO LIHIT
BE NO LIHIT
DE NO LIHIT
NO LIHIT
40
40
40
28
DE 90 72
NO LINIT
NO LIHIT
NO LIHIT
UNLIHITED
3,4 3.5
EEL 32 YES OUOTED
BOTH 150 YES NOT YET PUB
BOTH 40 1240 NOT YET PUBLISH
BOTH 29 NOT YET PUBLISH
BOTH 40 640 NO
BOTH 50 1300 TRIALS IN EUROP
BOTH 48 1249 DHSS
BOTH 60 1390 DHSS
PROF 9 79 NO
SEL 200 200 NO
BOTH lO0 100 YES
BOTH 300 1700 NO
BOTH 200 3000 NO
BOTH 300 SlO0 NO
BOTH 300 5700 NO
BOTH 300 SlO0 NO
BOTH 300 4200 NO
BOTH 300 110500 NO
BOTH 300 5400 YES
BOTH 300 7900 NO
BOTH 300/10200 NO
BOTH 38 170 YES OUOTED
BOTH 36 342 YES OUOTED
BOTH 38 494 YES OUOTED
BOTH 38 418 YES OUOTED
BOTH 39 646 YES OUOTED
BOTH 38 674 YES OUOTED
BOTH 80 640 NO
BOTH 90 1920 NO
BOTH O0 2240 HO
BOTH 80 250 HO
BOTH 39 NO
PROF 80 YES OUOTED
PROF 300 NOT CLINICAL
BOTH 220 7040 NOT YET PUBLISH
BOTH 90 1960 NO
BOTH 90 2161 NO
BOTH 40 520 NO
BOTH 64 64 HO
BOTH 90 2400 YES OUOTED
BOTH 55 55 NOT YET PUBLISH
BOTH 55 110 NOT YET PUBLISH
BOTH 30 900 YES OUOTED
BOTH 30 900 YES OUOTED
BOTH 60 1600 YES OUOTED
BOTH 90 2700 YES QUOTED
BOTH 50 1050 YES OUOTED
BOTH 39 722 YES
BOTH 76 1444 YES
SEL NOT YET PUBLISH
SEL 40 1200 NO
BOTH 160 YES QUOTED
BOTH lO0 NO
PROF 40 NO
PROF 40 NO
PROF 40 NO
PROF 29 NO
BOTH 72 NO
SEL 16 16 NO
BOTH 298 NO
BOTH 30 100 YES OUOTED
BOTH 30 100 YES OUOTED
BOTH 30 tO0 YES OUOTED
BOTH 30 100 YES OUOTED
PROF 144 NO
BOTH 144 NO
BOTH 20 NO
1500 YES NOT YET PUB
BOTH 12 NO
48
j. Fyffe et al. Review of large biochemistry analysers
NO lliST 4, 4.2a 4.2b 4.2c
AilliOTT VPS
2 PARALLEL
3 PERSPECTIVE
4 ENCORE II
$ 8PLAIT
6 HITACHI 7O4
HITACHI 705
ii HITACHI 737
? EASY
lO EPOS
II IRIS
12 EXREL
13 EXTRA
14 AIJS021
15 AUSI21
16 AUSO31
17 AUSI31
18 AU5041
19 AU5141
20 AU5061
21 AUS081
22 AS4
23 AS8
24 A6 ENZYHE
25 AS LIPID
26 HINI IDEAL
27 AS IDEAL
28 CX3
29 CX4
30 CX5
31 CX7
32 ANCA
33 CHEHILYRER
4 8Y61'EIM
5 PRIBA II
CORNIN6 580
37 CORNIN6 570
38 CORNIN6 EXPRESS
39 CPA
40 DACOS 2
41 DIHENBION 360
42 DIHENSION 760
43 6REINER 6-400
44 6REINER 6-450
45 TANOEH 2
46 TANDEH
47 6EHESIS 21
40 HOMNCH
49 HONARCH PLUS
50 EKTACHEH DT
51 EKTACHEH 700
52 PROORESS
53 SPECIFIC
54 HOVA DIOHED 1,1
55 NOVA iilOHEO 4,4
56 NOVA DIOHED 5,5
57 CODAS FARA
56 CODAS HIRA
59 ANBIDT
CHEH
61 RA-IO00
62 RA-2!
63 RA-500
64 DA-XT
65 SHAC II
66 8RA 2000
67 6Ell PROFILER
68 ULTROLAII
69 VITALAII 20O
EVERY RUN
DAILY
DAILY
EVERY RUN ZO TESTS
UNDER INVESTIOATION
DALLY REAGENT DLANK
DALLY REAGENT BLANK
DAILY REAGENT BLANK
HOST HONTHLY
EACH DATCH
HOST MEEKLY
AS FOR ERISIEPOS
HOST MEEKLY
HOST NEEKLY
HOST NEEKLY
HOST MEEKLY
HOST MEEKLY
HOST NEEKLY
HOST MEEKLY
HOST NEEKLY
HOST NEEKLY
9 HOURS
8 HOURS
8 HOURS
O HOURS
9 HOURS
6 HOURS
6 HOURS
UP TO NEEKS
UP TO 2 NEEKS
UP TO MEEKS
REAGENT DEPENDANT
DALLY
EACH BATCH
VARIES
ONCE PER DAY
ONCE/DAY
ONCE/DAY
NEEKLY
2 MEEKLY DEP ON CHEH
3 HONTHS
3 IITHS
2-3 HONTHS
2-3 HONTHS
2-3 HONTHS
2-3 HONTHS
24 HOURS
8 HOURS-7 DAYS
8 HOURS-7 DAYS
90 DAYS
90 DAYS
VARIES
VARIES
AUTOCAL
AUTOCAL
AUTOCAL
VARIABLE
VARIABLE
DALLY
1-4 NEEKS
2 NEEK6
2 ilEEKS
2 MEEKS
2 NEEKS
96 SAILES
SEE BIIAC IIIRA-IO00
MITH lIEN REARENTS
N/DA
VMIES
AOENT CALIDRATORS
BERUN DARED
BERUH BASED
RERUH
EITHER
EITHER
EITHER
EITHER
RERUN
EITHER
EITHER
EITHER
EITHER
EITHER
EITHER
EITHER
EITHER
EITHER
EITHER
EITHER
EITHER
EITHER
EITHER
EITHER
EITHER
EITHER
EITHER
EITHER
EITHER
EITHER
EITHER
RERUN
BERUH
RERUH
NONE RECOHHENDED
RERUH
RERUN
SERUH
RERUN
RERUN
EITHER
EITHER
RERUN
SERUN
SERUN
SERUH
EITHER
EITHER
EITHER
BERUH
RERUN
EITHER
EITHER
NOVA CALIDRANTS
NOVA CALIDRANTS
NOVA CALIDRANTS
EITHER
EITHER
RERUN
EITHER
EITHER
EITHER
RERUN
EITHER
RERUN
RERUN
EITHER
N/DA
AOUEOUS
YES
YES
YES
YES
YES
YES
YES
YES
NO
YES
YES
YES
YES
YES
YES
YES
YES
YES
YES
YES
YES
NO
NO
NO
NO
YES
YES
YES
YES
YES
YES
YES
YES
YES
YES
YES
YES
YES
YES
YES
YES
YES
YES
YES
YES
YES
YES
KODAK CAL]DRATORS
KODAK CAL]BRATORS
YES
YES
NO
YES
YES
YES
YES
YES
YES
YES
YES
YES
YES
N/DA
YES
/2
/2
/2
16
/3
/5
/5
/5
/2
/6
10
16
20
11
/I1
11
11
11
11
11
/11
14
16
1116
DEP ON CALIBRATOR
1/8
1/8
1/2
1/2
H/DA
N/DA
0/6
1/6
3/5
3/5
1/6
1/6
1/6
1/6
1/5
1/6
I/6
2/4
2/4
1/6
1/6
ALL INCLUDED IN PACk[
ALL INCLUDED IN PACK
ALL INCLUDED IN PACK
016
0/9
1/6
1/6
1/6
2
1/??
N/DA
1/4
49
J. Fyffe et al. Review of large biochemistry analysers
NO INST 5.1 5.2 5.3 .4 6.1 6.2a 6.2b
ABBOTT VPS
2 PARALLEL
3 PERSPECTIVE
ENCORE II
$ SPIRIT
6 HITACHI 704
HITACHI 705
6 HITACHI 737
? EA6Y
10 EPOS
11 ERIS
12 EXBEI.
13 EXTRA
14 AUS021
15 AUSI21
16 AU5031
17 AU5131
IB AU5041
1 AUSI41
20 AUS061
2! AUSOOI
22 AS4
23 ASS
24 A6 ENZYRE
25 AS LIPID
26 HIN! ]DEAL
27 AS ]DEAL
28 CX3
29 CX4
30 CX5
31 CX7
32 AGCA
33 CHEHILYGER
34 SYSTEH4
35 PRIBHA
CORNIN6 590
37 CORNIN6 570
30 CORNIN9 EXPRESS
39 CPA
40 DAC06 2
41 DIHENSIOH 300
42 DIHENB|OH 760
43 6REINER 6-400
44 6SEINES 6-450
45 TANOEII 2
46 TANDEN 3
47 BENESIS 21
40 HOHARCH
49 HORARCH PLUS
50 EKTACHEN DT
51 EKTACHEH 700
52 PROORE88
53 SPECIFIC
54 NOVA DIORED Ill
55 NOVA BIOHED 4,4
YES YES
YES YES
YES YES
YES YES
HE BELIEVE YES
YES
YES
YES
YES
YES
YES
YES
YES
YES
YES
YES
YES
YES
YES
YES
YES
YES
YES
YES
YES
YES
YES
YES
YES
YES
YES
YES
YES
YES
YES
YES
YES
YES
YES
YES
YES
YES
YES
YES
YES
YES
YES
YE6
YES
YES
YES
YES
YES
YES
YES
YES
YES
YES
YES
HOT OUOTED YES
NOT OUOTED YES
YES YES
YES YES
YES YES
YES YES
YES YES
YES YES
YES BY REPLCUP NO UIE
YES BS 600 YES 40 HINS
YES D O00 YES 24 HlNS
YES DS 900 YES NO U/E
HE BELIEVE UHKHOHH YES 25 HINS
YES D8 900 YES 25 HIN8
YES 98 800 YES 25 HINS
YES DE 900 YES 30 HIM
NO UIE
22 HIHS
12 HING
NO U/E
15 RING
12 NlNS
12 HINS
YES BS 4743 YES
YES BS 4743 NO UIE
YES DS 4743 YES
YES ? DS 4743 YES
YES DS 4743 YES
YES ? DS 4743 YES
YES DS 4743 YES
YES BS 4743 YES
YES BS 4743 YES
YES D$ 4743 YES
YES BS 4743 YES
YES BS 4743 YES
YES ? BS 4743 YES
YES YES
YES YES
YES YES
YES YES
YES YES
YES YES
YES YES
YES YES
YES YES
YES YES
NOT AVA]L DS 5724 YES
YES NO
YES DY REPLCUP 75 HINS
YES YES 25 fliNG
YES TESTED JSl YES 60 HINS
YES TESTED GI YES 15 fliNG
YES IEC 601 YES NO UIE
YES YES NO U/E
YES YES 32 HINS
ALHAYS OH 8 HINS
NO U/E NO UIE
15 HINS 11 HINS
15 HINS 11 HIN8
15 HINS 11HIHG
IHR POH OF 19 HINS
IHR POH OF 18 HINS
tHR POH OF 18 RING
IHR POH OF 19 HINS
IHR POH OF lO HINS
IHR POH OF 19 HINS
IHR POH OF 19 HINS
tHR PON OF 19 H]NG
ALHAY8 ON
ALHAYS ON HIN
ALNAYS OH 1HIH
ALNAYS ON HIN
ALHAYS ON
ALNAYS ON HIN
ALNAYS ON (1 HIH
ALNAYS ON NO U/E
AL#AYS ON <13 HINS
ALNAYG ON (1 HIH
NO U/E NO U/E
60 HIN 30 HIN
10-20 SINS
L5 HIHS
5 fliNG
NO UIE
NO U/E
12 HINS
YES
YES
YES
YES
YES
YES
YES
YES
YES
YES
YES
YES
YES
YES
YES
UH DEVELOP YES
UN DEVELOP YES
YES
YES
YES
YES
YES
YES
YES
YES
YES
YES
YES
YES
YES
YES
YES
YES
YES
YES
YES
YES
YE6
YES
YES
YES
YES
YES
DS 800
DS 800
DS 900
DS 600
BS 5724
98 5724
BS 5724
YES 6 HINO HIHS
YES HINS HINS
YES 15 HIN6 15 RING
YES 12 SINS 12 H]NG
YES 12 HINS 12 HINS
YES 12 HINS 12 HINS
YES 13 HIN6 13 HIHS
YES 6 HINS 6 HINS
YES 6 HING 6 HINS
YES ALNAY8 ON 9 fllN8
YES ALNAYS ON 10 H|NS
YES ALHAYS OH 9 HINS
YES ALNAYS ON 9 HINS
YES NO U/E NO U/E
YES HO U/E NO U/E
56 NOVA BIOHED 5+5 YES YES
57 CODAS FARA YES YES
59 CODA6 HIRA YES YES
5 ASSIST YES YES
60 CHEfl YES YES
61 RA-IO00 YES YES
62 RA-2X YES YES
3 RA-500 YES YES
64 RA-XT YES YES
65 9HAC II YES YES
66 81iA 2000 YES YES
67 9EH PROFILER YES NO
66 ULTRoLAD N/DA YES
69 VITALAD 200 YES YES
YES
YES
YES
YES
YES
YES
YES
YES
YES
YES
YES
YES
YES
? YES
YES
96 5724 YES
B8 5724 YES
YES
YES
YES
YES
YES
YES
YES
YES
YES
N/DA YES
!i900 4743 YES
NO U/E NO U/E
12 HINS HIHS
12 HINS HIN8
NO UIE NO UIE
30 HINS 15 HIN
ALHAYS ON 3.5 HINS
ALHAYS ON 3.5 HIHS
NO UIE NO UIE
ALHAYS OH 3.5 HIN
35 HINS 20 HIHS
ALLAY8 ON 5* HINS
NO UIE NO U/E
NIDA N/DA
NO UIE NO UIE
5O
J. Fyffe et al. Review of large biochemistry analysers
NO INST 7.1 7,2 7.3 7.4 7,5a 7,5b 7,5c 7,6
ABBOTT VPS
2 PARALLEL P2o15,1o30
3 PERSPECTIVE P1o30
4 ENCORE II PIo20A BES-IOL/I)AY
5 SPIRIT PIoI3A
6 HITACHI 704 PIo20A BE+DR
HITACHI 705 PIo20A BE+DR
8 HITHI 737 PIo30A DE+DR
9 EASY
10 EPt
II ERIE BE6.2LIHR
12 EXSEL BE6, 2L/HE
13 EXTRA PV BEI2,4L/HR
14 AU5021 P4,KV BE3OL/HR+DR
15 AUSI21 P4,SK9 BEOL/HR+DR
16 AU5031 P6,0KV BE4$L/HR+OR
17 AUSI31 P6, OV BE45L/HR+BR
18 AU041 P7,0KV BE6OL/HR+DR
19 AUSI4! P7,0KV DE6OL/HR+DR
20 AU5061 PII,OKV BEOL/HR+I)R
21 AU5081 PI4,0K9 BEI2OL/HR+I)R
22 AS4
23 A68
24 AS ENZYHE
25 AS LIPII)
26 HINI IBEAL
27 AS IBEAL
28 CX3
29 CX4
30 CX5
31 CX7
32 ASCA DE+DR
3 CHEHILYSER
34 SYSTEH4
35 PRISHA II
36 CORNIN6 560
7 CORNIH6 570
38 CORNIN6 EXPRESS
39 CPA
40 DACO$ 2
41 DIHEHBION 360 P1o13,
42 DIHEN8IOH 760 Plol
43 OREIHER 6-400
44 OREINER 6-450
45 TAHBEH 2
46 TANBEH 3
47 6ENESIS 21
48 HONARCH
49 HONARCH PLUS P3013A
50 EKTACHEH DT P2013A
51 EKTACHEH 700 PIo20A
52 PRO6RESS
53 CIFIC
54 HOVA BIOHED 1+1
55 NOVA DIOHED 4+4
NOVA BIORED 5+5
57 CODAS FARA DE 5L/BY
58 CODAS HIDA BE 5L/BY
59 ASSIST PIoI3A
60 CHEN PIo3OA
61 RA-IO00 PIo20A
62 RA-2X P2020A
3 RA-500 PIo20A
64 RA-XT PIo20A
65 SHAC II PIo30A
66 8RA 2000 PIo30A
67 6EH PROFILER P4o1
69 ULTROLAD
69 VITALAD 200
BENCH 1.5ol 60 2BY 2 INCL N/DA
FLOOR 2.300,6/+ 1050 BOY 2 INCL 16
FLOOR 2,001,5// 940 5BY 2 INCL 5
FL+DE 2.001,5 263 INK 2 INCL 32
BENCH 2.001.5 96 4BY 2 INCL 0
FLOOR 2ol 350 2NK8 2 INCL 12
FLOOR 2ol 350 INK 2 INCL 60
FLOOR 3Ol,5 600 2NKS INCL 14
FLOOR O,6SqH 170 2BY 2 IHCL 0
BENCH ISqH 140 2BY INCL
FLOOR 2.2SqH 500 31)Y 2 INCL 15
FL+DE 4.SSqH 740 INK 3 INCL
FLOOR 6.0Sq 11OO iNK 3 INCL 0
FLOOR 2.803.2++ 1195 INK [NCL 0
FLOOR 2.603.2++ 1195 INK 3 INCL
FLOOR 3.203.2/+ 1585 INK INCL 0
FLOOR 3.203.2+ 1585 INK :3 INCL 0
FLOOR :3.903.2++ 1685 INK ]NCL 0
FLOOR :3.903,2++ 1685 INK 3 INCL
FLOOR 3.203.2/+ 2596 INK 3 INCL 0
FLOOR 3.903.2// 2765 INK 3 INCL 0
BENCH 1,4o0,7 151 INK INCL 46
FLOOR 1.5t0.8 296 INK 2 INCL 60
FLOOR 1.500.9 296 till( INCL 2:3
FLOOR 1.500,8 296 INK 2 INCL 8
FLOOR 2.900.8 447 iNK 2 INCL tO
FLOOR 3.000.9 592 INK 2 ]NCL 16
FLOOR 0.700.8 159 INK ]NCL
FLOOR 1.200.6 205 INK 2 INCL 0
FLOOR 1.600.9 250 INK 2 INCL 0
FLOOR 1.9o0.9 364 INK 2 INCL 0
BENCH 75 YES 0
FLOOR 2.4i0.7 NII)A 5BY 5 INCL 2
BENCH :3.000.5 100 20Y 5 250 20
P3PHABE DI32L/HR@E FLOOR 12 SqH 700 INK 2 IHCL
P2015A DE+DR+TH+CA FLOOR 2o1,5 450 INK INCL 0
I)E/TN (37 FLOOR 1ol 30 2-3BY 2 INCL 0
BENCH 0.501.5 2-3BY INCL
PIiI3A BENCH 0.82i0.75 55 3BY INCL 0
PIJI6A BEII.4L/HR (:32C FLOOR 1,1o1,1 339 SOY 2 INCL 10
FLOOR 0,7ol,3 200 4BY 2 IHCL 0
FLOOR 0,702,6 400 4BY 3 INCL 0
DE35LIHR+DR FLOOR 3.502 515 INK 2 INCL 11
BE6OL/HR/OR FLOOR :3,5o2 515 INK INCL 0
BEI20LIHR+DR FLOOR ,5o3,5 1030 INK 2 IHCL 0
BEIOOLIHR+DR FLOOR 5,0o3.5 1545 INK INCL 0
BE7LIHR OIILI4HR FLOOR 1.501,5 200 2,SOY 2 INCL
FLOOR 2,001,5 295 3BY INCL 12
FL+DE 5.0tl.5 620 4BY 2 INCL 2
BENCH 2,0Ol 22,9 IDY ]NCL 26
FLOOR 2.5i1,5 500 INK 2 INCL 3
(32C BOTH 1.001.5 126 :3BY INCL 25
BOTH 1.0il.5 120 3BY 2 INCL
BENCH 0,900,6 02 IOY N/AV 150 41
BENCH 0,900,6 92 IDY NIAV 150 2
BENCH 0,900,6 62 tDY H/AV 150
BENCH 3,002,6 165 4BY IHCL 41
BENCH 1,301,2 60 4BY [NCL 39
BENCH l.OJl,O 50 3BY 2 INCL 0
FLOOR 3.0o2.0 700 iNK 2 ]NCL
BENCH 2.0o0.6 125 INK 2 INCL 120
BENCH 4.0o0.6 280 2NK 2 INCL
BENCH 4.0o0.6 125 iltK 2 INCL 14
FLOOR 4.0t1.0 140 2NK 2 INCL
FLOOR 3.0o3.0 900 3NK 2 INCL 24
FL+BE 3.0t3.0 1200 4ilk 2 [NCL 14
BENCH 3 9q H 30 4BY 2 INCL 0
NIDA 1,5 40 IDY 2 N/DA 0
BENCH 37 IDY 1-3 INCL 0
51
j. Fyffe et al. Review of large biochemistry analysers
NO INST 8,1a 8.1b
ABBOTT VPS NIDA N/DA
PARALLEL 30 HRIYR HR
3 PERSPECTIVE 20 HR/YR HR
ENCORE
5 SPIRIT 3JO.SDY/YR 30 liINo
6 HITACHI 704 20 HR/YR 20 BINS
HITACHI 705 20 HRIYR 20 NINS
0 HITACHI 737 20 HR/YR 30
9 EASY N/DA N/I)A
10 EPOS 6 HR/YR
11 ERIS 13 HRIYR
12 EXSEL 20 HR/YR
13 EXTRA 26 HR/YR
14 AU5021 16 HR/YR N/DA
15 AUSI21 16 HRIYR
16 AU5031 16 HR/YR
17 AUS131 18 HRIYR
18 AU5041 20 HR/YR
19 AU5141 20 HR/YR
20 AU5061 24 HR/YR
21 AU5081 28 HR/YR
22 AS4 DY/YR 5 lilNS
23 ASS DY/YR 5 lilNS
24 AS ENZY DYIYR 5 liINS
25 AS LIPID DY/YR 5 liINS
26 lilNl IDEAL I)Y/YR 5
27 AS IDEAL DY/YR 5 liINS
28 CX3 DY/YR HINS
29 CX4 DYIYR N/DA
30 CX5 DY/YR N/DA
31 CX7 DY/YR N/DA
32 ARCA DY/YR SHINS
33 CHENILYSER 2 DY/YR 25 liINS
34 SYSTEli4 2 DY/YR 25 lilNS
35 PRIMA II 40 HR/YR 30 IIINS
36 CORNINO 590 26 HR 30 liINS
37 CORNIN6 570 N/I)A 30 liINS
38 CORNIN6 EXPRESS 16 HR/YR 15
39 CPA 12 HRIYR liIN
40 6ACOR 20 HR/YR 12
41 DIHENSION 380 12 HR/YR 10 liINS
42 OIHENOION 760 12 HR/YR 10 lilNS
43 6REINER 6-400 DYIYR
44 6REZNER 6-450 2 I)Y/YR
45 TANDEli 2 2 DY/YR
46 TANDEN 3 2 DY/YR
47 6ENESIS 21 2 DY/YR
49 NONARCH 2 I)Y/YR 10
49 NONARCH PLUS 2 4 DYIYR 20 lilNS
50 EKTACHEH I)T HR/YR 5 liINS
51 EKTNCHEN 700 I)Y/YR 10 BINS
52 PROORE88
5,1 SPEClFZC
54 NOVA BIOREI) 1.1
55 NOVA DIOHEB 4*4
NOVA BIOHED
57 CORA9 FARA
56 CODAS HIRA
59 ASSIST 0 15 HIN
60 CHEH DY/YR (5 liIN
61 RA-IO00 0 15 liIN
62 RA-2X 0 215 HIN
63 RA-500 0 15 HIN
64 RA-XT 0 15 HIN
d 8HAC II 4 DY/YR HR
66 BRA 2000 DYIYR HR
67 6EH PROFILER 2toO.SOY 0
66 ULTRoLAD
69 VITALAD 200 2 DYIYR 10 HINB
6,2 6.3 8,4 6.5
NIDA NO 6liTH
LEVELS 9.3% SPARES IYR
LEVELS 6.3% NO IYR
FULL 46 PH 1836 8.6% NO IYR
FULL 3500 10% SPARES IYR
FULL 6360 3 LEVELS AVAIL 9,6% NO tYR
FULL 6420 3 LEVELS AVAIL NO IYR
FULL 11560 LEVELS AVAIL 9% NO IYR
SPARES IYR
NO IYR
SPARES IYR
SPARES IYR
SPARES
SPARES IYR
SPARES IYR
SPARES IYR
SPARES IYR
SPARES 1YR
SPARES LYR
SPARES IYR
SPARES IYR
FULL 4652 SPARES 1YR
FULL 6496 SPARES IYR
FULL 9094 SPARES IYR
FULL 7295 SPARES IYR
FULL 11947 SPARES IYR
FULL 15389 SPARES IYR
FULL 4000 SPARES IYR
N/DA SPARES IYR
N/DA SPARES IYR
N/I)A SPARES IYR
NO IYR
FULL 4500 NO IYR
FULL 2500 NO IYR
FULL 14000 5 LEVELS AVAIL SPARES IYR
FULL 11200 SPARES
FULL 7500 SPARES IYR
FULL 3000 SPARES IYR
NO
SPARES
SPARES
NO
SPARES
NO
NO
NO
SPARES
SPARES
SPARES
SPARES
SPARES
SPARES
SPARES
SPARES
SPARES
SPARES
SPARES
SPARES
SPARES
SPARES
SPARES
SPARES
SPARES
SPARES
SPqES
SPARES
SPARES
SPARES
SPARES
NO
NO
SPARES
SPARES
SPARES
SPARES
FULL 2600
FULL 6000 2 LEVELS AVAIL
FULL 6200 LEVELS AVAIL
FULL 6200 4 LEVELS AVAIL
FULL 12000 4 LEVELS AVAIL
FULL lO000 LEVELS AVAIL
FULL 4500 Pli 550
FULL 5500 PH 540
FULL 12700 PH 1080
FULL 680
FULL D500
FULL 4200
FULL 2600
NO I)ATA
FULL 9950
FULL 3647
FULL 8546
FULL 347
FULL 5968
FULL 17244
FULL 20063
FULL 1500 PH 480
PH 3.5% FULL 9,5% NO
FULL 9.5% NO
6% NO
6% NO
9% NO
NO
NO
ISIC 6% FULL IOZ NO
10% NO
NO
NO IRE IY NO
NO ISE lY NO
6% SPARES I-2YR SPARES
6% SPARES I-2YR SPARES
NO IYR HO
NO IYR NO
NO IYR NO
NO IYR NO
SPARES IYR SPARES
SPARES IYR SPARES
SPARES IYR SPARES
SPMES IYR SPMES
SPARES IYR SPARES
IYR NO
IYR NO
IYR YES
IYR YES
IYR YES
IYR NO
IYR NO
NO IYR NO
SPARES IYR SPARES
NO IYR NO
NO IYR NO
NO IYR NO
NO IYR NO
SPARES IYR SPARES
EPMES IYR SPARES
IYR NO
IYR NII
IYR NO
6.6
7YR
7YR
7YR
)TYR
)7YR
7YR
7YR
7YR
)IOYR
)IOYR
)IOYR
)IOYR
)IOYR
)IOYR
)IOYR
)IOYR
)IOYR
)IOYR
)IOYR
>IOYR
>IOYR
7YR
7YR
7YR
7YR
7YR
7YR
7YR
7YR
7YR
7YR
IOYR
JOYS
IOYR
IOYR
7YR
7YR
7YR
INDEF
INDEF
IOYR
IOYR
>IOYR
)tOYS
)IOYR
)tOYS
IOYR
IOYR
IOYR
tOYS
IOYR
>SYR
>SYR
)7YR
>7YR
>7YR
>TYR
)7YR
7YR
7YR
7YR
7YR
7YR
7YR
7YR
7YR
INDEFF
N/DA
IOYR
Eli
Ell
EH
En
Eli
EH
EH
EH
EH
Eli
EH
EH
EH
EH
EH
EH
Eli
En
EI
Eli
Ell
Ell
Eli
EH
Ell
Ell
Ell
EH
Ell
EH
Eli
Eli
EH
Eli
52
j. Fyffe et al. Review of large biochemistry analysers
NO INST 9.1i 9.1b 9.2
ABBOTT VPS NO YES NO
2 PARALLEL YES YES
3 PF.RSPECTIVE YES YES
4 ENCORE II NO YES NO
5 8P|RIT YES INCL YES
6 HITACH! 704 YES YES YES
HITACHI 705 YES YES NO
8 HITACHI 737 YES YES YES
9 EASY NO NO NO
lO EPI)S NO YES NO
l! ERIS YES YES YES
12 EXBEL YES YES YES
i3 EXTRA YES YES YES
14 AU5021 YES YES YES
15 AU5121 YES YES YES
16 Na031 YES YES YES
17 AU513! YES YEs YES
18 AU5041 YES YES YES
19 AU$141 YES YES YES
20 AIJS061 YES YES YES
21 AU5001 YES YES YES
22 AS4 NO YES YES
23 AS8 NO YES YES
24 AS ENZYHE NO YES YES
25 AS LIPID NO YES YES
26 HINt IDEAL YES H/APP YES
27 AS IDEAL YES N/APP YES
26 CX3 YES H/APP YES
29 CX4 YES N/APP YES
CX5 YES N/APP YES
31 CX7 YES H/APP YES
32 ARCA YES YES YES
33 CHEHILYSER YES N/APP YES
34 SYSTEH4 NO YES HO
35 PRISM II YES YES
3 CORNINO 580 YES YES NO
37 CORNINO 570 YES YES NO
38 CORNINO EXPRESS YES YES YES
39 CPA YES N/APP YES
40 DACOS 2 YES N/APP YES
41 DIHENBION 380 YES YES YES
42 DIHENBION 760 YES YES YES
43 MEINER 6-400 NO YES YES
44 6REINER 6-450 NO YES YES
45 TANDER 2 YES YES YES
46 TANOEH 3 YES YES YES
47 6F.NESIS 21 YES YES NO
40 NONMCH NO YES NO
47 HONARCH PLUS 2 YES YES YES
50 EKTACHEH DT NO NIAV NO
51 EKTACHEH 700 YES N/AV YES
52 PROGRESS YES YES
53 SPECIFIC YES YES
54 NOVA DIOIIED !.1 HO H/AV HO
55 NOVA B|OHED 4*4 NO N/AV NO
NOVA DIOHE]) 5*5 HO H/AV NO
57 CODAS FMA YES YES
56 CODAS HIRA YES YES
I CHEH YES STD YES
61 DA-IO00 NO YES NO
&2 RA-2X YES ST]) YES
63 RA-500 NO YES NO
RA-XT YES STD YES
65 BHAC I! YES STD YES
8RA 2000 YES ST]) YES
67 6EH PROFILER YES YES YES
ULTROLAD H/DA N/DA HIDA
69 VITALAB 200 YES YES NO
9.2b 9.3i 9,3b 9,4i 9.4b 9,4c
YES H/AV
lO00O
15000
YES 30 R/CUP flAX 3500
INCL 200 PID INCL
YES 400 PT 54RIPT 6t300C DEP ON DATA flANA6ER
YES OC 30 POINTIISODAY DEP ON DATA flANASER
YES Ld)O PT OC 6,30/30DAY DEP ON DATA HANA6ER
N/AV 300 PT VARIES
YES NO VARIES
YES 1274 PT HTH OC VARIES
YES 10000 PT 2 HTHS OC VARIES
YES t0000 PT HTHS OC VARIES
YES 4000 PT 2HTH 6C VARIES
YES 4000 PT 2 HTHS OC VARIES
YES 4000 PT 2 HTHS OC VARIES
YES 4000 PT HTH8 OC VARIES
YES 4000 PT 2 HT OC VARIES
YES 4000 PT 2 HTHS OC VARIES
YES 4000 PT 2 HTHS OC VARIES
YES 4000 PT HTHS OC VARIES
N/APP 76 DEP OH DATA flANA6ER
H/APP 76 DEP ON DATA HANA6ER
N/APP 76 DEP ON DATA flANA6ER
N/APP 76 DEP ON DATA HANAOER
H/APP 76 DEP ON DATA flANA6ER
NIAPP 76 DEP ON DATA HANA6ER
H/APP H/DA DEP ON DATA flANA6ER
N/APP N/DA DEP ON DATA HANAOER
H/APP H/DA DEP OH DATA HAHA6ER
NIAPP NIDA DEP OH DATA HAHASER
YES UNLIHITED REHOV DISC
NIAPP 490 H/APP
YES H/DA 300
2500 NON 200OBLATE67
YES 900 SYRTEH DEPEHD
YES 1000 YES
YES 360 YES
H/APP 126 PT 2560RIPT 30OC DEP ON DATA HANA6ER
HIAPP 1600P 600006 CI2HK DEP ON DATA flANA6ER
YES 300 PT 5000 PT 40000 OC PT
76 PT/3120 RES NO NO H/AV
YES YES
YES YES
364PT 22REIIPT 30 OC YES YES
NO YES
NO YES NIAPP
NO YES HIAPP
NO YES N/APP
YES NO
YES YES
YES YES
YES YES
YES YES
YES YES
YES YES
YES YES
YES YES
YES YES
YES YES
YES YES
YES YES
YES YES N/APP
YES YES NIAPP
YES YES N/APP
YES YES N/APP
YES YES N/APP
YES YES N/APP
YES YES N/APP
YES YEB N/APP
YES YES NIAPP!
YES YES N/APP
YES
YES H/AV
NO N/AV
YES
YES N/AV
NO N/A
NO NIAV
YES H/AV
NO
NO
NO
YES
YES
YES
YES
NO
NO
YES
YES 300 PT 5000 PT 40000 OC PTB NO YES
YES DAY 4000 PT YES YES
YES DAY 4000 PT YES YES
YES DAY 4000 PT YES YES
YES DAY 4000 PT YES YES
YES OC 20 DAYS 1000 RECORDS YES YES
YES NONE 20000 PIDIRES OCSODY YES YES
YES 20000 PID/RES OCSODY N/APP YES YES
HIAV 15 RESULTS NIAVV YES NO
NIAV 400 PT RE8 TO OC RE N/AV NO YES
260 PID 15006 OCSIDY NO YES
64 PID 5006 OC 31 DY NO YES
NIAV NO N/AV
N/AV NO H/AV
NIAV NO NIAV
VARIADLE
VARIABLE
NO 0 0 HO NO
STD 2000* 6Ti) YES YES
YES 0 2000, YES YES
STD 2000, STD YES YES
YES 0 2000, YES YES
6TD 2000* 6TD YES YES
8TD 50(0 BTD YES YES
STD 5000 ST]) YES YES
YES IOK FILES 401RES/FIL YES
NIDA H/DA NIDA NIDA N/DA
YE6 12 PID/RE8 iiC INOEFF YES NO
N/AV
1500
1500
HO
HIDA
53
j. Fyffe et al. Review of large biochemistry analysers
54
NO INST ?.Sa 9.5b 9.6a 9.6b 9.7a
ABBOTT VPS FI](ED YES
2 PARALLEL USER DEFF
3 PERSPECTIVE USER DEFF
4 ENCOI II NO
5 SPIRIT FIXED
6 HITACHI 704 FIXED
7 HITACHI 705 FIXED
S HITACHI 737 FIXES USER DEFF
9 EASY NO YES
10 EPOS NO YES
11 ERIE YES YES
12 EXSEL YES YES
13 EXTRA YES YES
14 AU5021 YES YES
15 AU5121 YES YES
16 AU5031 YES YES
17 AU5131 YES YES
IS AU5041 YES YES
19 AU5141 YES YES
20 AU5061 YES YES
21 AUS081 YES YES
22 AS4 NO YES
23 AS8 NO YES
24 AS ENZYNE NO YES
25 AS LIPID NO YES
26 NINI IDEAL NO YES
27 AS IBEAL NO YES
28 CX3 NO YES
29 CX4 NO YES
30 CX5 NO YES
31 CX7 NO YES
32 ASCA NO
33 CHENILYSER DEFF N/AV
34 SYSTEII4 NIAV F| XED
35 PRISM II YES
6 COllINS 580 USER DEFF USER DEFF
37 COflNINO 570 YES YES
8 CORNINO EXPRESS N/DA YES
39 CPA FIXEB YES
40 BACOB 2 USER DEFF NIAPP
41 BIIIENBION 3410 FIXED USER DEFF NOINO
42 BIIIENOION 760 FIXED USER DEFF
43 6REINER 6-400 YES YES
44 611EINER 6-450 YES YES
45 TANBEII 2 YES YES
46 TANDEll 3 YES YES
47 6ENESIB 21 FIXES USER BEFF
48 NONARCN F!XEB USER DEFF
49 NONARCN PLUS 2 USER DEFF USER BEFF
50 EKTACHEli BT FIXED NIAV
5! EKTACHEll 700 USER DEFF NIAV
52 PROGRESS USER DEFF
53 SPECIFIC USER DEFF
54 NOVA BIONED 1+1
55 NOVA BIOIIEB 4+4
56 NIWA BIOHED 5+5
57 CODAS FARA FIXED
811 COBAB NIRA FIXED
59 ASSIST NO NO
60 CHEN YES STD
61 RA-tO00 NO YES
62 RA-2X YES STD
63 RA-500 NO YES
64 RA-XT YES STD
65 II YES STD
BIIA 2000 YES STD
67 liEN PROFILER USER DEFF YES
J ULTNOLAB NIDA NIBA
69 VITALA 200 FIXES VARIABLE
YES
VARIABLE
UER DEFF YES/YES
USER DEFF NO/NO
NO YES NO
YES/YES NO
YES/YES NO
NO YES/YES NO
YES/YES/?? YES/YES/?? ?YES/YES/???
YES/YES NO/NO/NO
YES/YES NO
YES/YES YES/YES YES/YES/YES
NO/NO YES NO
NO/NO YES NO/NO/NO
YES/YES YES NO/NO/NO
YES/YES YES NO/NO/NO
YES/YES YES NO/NO/NO
YES/YES YES NO
YES/YES YES NO/NO/NO
YES/YES YES NO/NO/NO
YES/YES YES NO/NO/NO
YES/YES YES NO
YES/YES YES NO/NO/NO
YES/YES YES NO/NO/NO
YES/YES YES NO/NO/NO
NO YES NO
NO YES NO
NO YES NO
NO YES NO
NO YES YES
NO YES YES
NO YES NO
NO YES YES
NO YES YES
NO YES YES
YES/YES NO
NO NO NO
YES/YES YES/YES/YES
YES YES NO
YES/YES YES NO/NO/NO
YESlYEB YES NO
NO YES NO
YES/YES NOT/oP NO/NO/NO
YES/YES NO
NO/NO YES/YES NO
NO/NO YES/YES NO/NO
NO/NO YES/YES NO
YES/YES YES/YES YES/YES/YES
YEBIYEB YEBIYE8 YEBIYEBIYE8
NO YES NO
NO YES NO
YES YES YES
NO/NO NIAV NO
YES/NO NIAV NO
YES/YES YES
YES/YES YES
9,7b 9.8a 9,8b
NO YES N/AV
YES YES VARIABLE
YES YES 6000 NAX
POSSIBLY YES VARIES
?YES/YES/??? YES MOT KNOMN
YES/YES/YES YES DEP ON REOU]RE
YES/YES/YES YES DEP ON REOUZRE
YES/YES/YES YES DEP ON REOUIRE
YES YES NIDA
YES YES M/DA
YES YES N/DA
YES YES N/DA
YES YES N/BA
YES YES N/DA
YES YES N/DA
YES YES N/OA
YES YES N/DA
YES YES NIDA
YES YES N/DA
YES YES N/DA
YES YES N/DA
YES YES VARIES
YES YES VARIES
YEB YES VARIES
YES YES VARIES
N/APP YES VARIES
NIAPP YES VARIES
YES YES VARIES
NIAPP YES VARIES
N/APP YES VARIES
N/APP YES VARIES
YES NESOTIABLE
NO YES N/AV
NO YES NIA9
YES RAX '5000
YES YES SYSTEH DEPEND
YES YES SYSTEH DEPEND
YES YES BYSTEN DEPEND
YES YES TBA
YES YES TOTAL 'I700
YES/YES/YES YES VARIES
YES/YES/YES YES VARIES
YES/YES/YES YES VARIES
YES/YES/YES YES VARIES
YES/YES/YES YES VARIES
YES/YES/YES YES VARIES
YES YES SYSTEH DEPEND
YES YES SYBTER DEPEN
YES YES SYSTEN DEPEND
NIAV YES VMIE8
N/AV YES VARIES
YES VARIES
YES VARIES
NO
NO
NO NO
YEB BTD
NO YES
YES BTD
NO YES
YES STD
YES STD
YES STD
YES/YES YES
M/DA N/DA
NO/NO YES/YES
NO YES VARIES
NO YES VARIABLE
NO NO NIAV NIAV
NO STB YES BOFTNARE COSTS
NO ACP STATISTICS YES 80FTMARE COSTS
ACP STATISTICS 8TD YES 80FTHARE COSTS
NO ACP STATISTICS YES 81)FTMARE COSTS
ACP STATISTICS STD YES 6(WTNARE COSTS
NO NO' YES SOFTNARE COSTS
NO NO YES SOFTNARE COSTS
YES YES YES
NIDA MIDA MIDA NIDA
NO YEBIYEBIYEB YES NIDA
j. Fyffe et al. Review of large biochemistry analysers
NO INBT 10,1 10,20 I0,0 10,3 U/E 10,3 LFT 10,380H IO,3CARD 10,16C 10,3 6LU 10,3 AU.
ADDOTT VPR 22000 NI&V 4000 NO U/E N/DA H/DA N/DA N/DA N/DA N/DA
2 PARALLEL 217500 INCL INCL 11,67 13,06 3,97 11,23 12,27 2,t0 54,20
3 PERSPECTIVE 115000 INCL INCL 9.28 16.07 4,71 14,26 12,35 1,40 56.69
4 ENCORE II 49500 NIA 2-5000 NO UIE 16,63 10,50 14.49 12.42 NIDA NO UIE
5 SPIRIT 35000 INCL INCL 23,45 17,31 13,30 18,44 25,13 N/DA 97,63
6 HITACHI 704 49000 10000 DEPENDS 21,40 25,45 14,29 13,29 31,50 2,73 96,97
HITACHI 705 APP35000 12300 DEPENDS 25.90 24.90 13.80 13.40 30.70 5.11 96.90
8 HITACHI 737 129000 INCL INCL 20,56 22,11 12,53 12,66 20,67 4,46 91,62
9 EASY 37500 INCL VARIES 18%00 375,00 196,00 189.00 249.50 22,00 930,90
10 EP08 37500 HIAV VARIES NO UIE 11,5 6,20 5.70 14,33 2,00 NO UIE
11 ERIS 80000 16500 VARIES 10,78 15,49 10,44 7,65 17,28 3,42 56,67
12 EXL 1504)00 INCL INCL 10.76 11.50 6,20 5.70 14.33 2.00 44.72
13 EXTRA 180000 INCL INCL 10,78 15,48 10,44 7,65 17,29 3.42 56,60
14 AU5021 165000 10000 INCL 9,04 14,00 9,00 7,20 15,20 1,60 46,72
15 AU5121 165000 10004) INCL 9,04 14,08 %00 7.20 15,20 1.60 46,72
16 AU5031 210004)0 10000 IHCI. 9.04 14,09 9 O0 7.20 15,20 1,60 46,72
17 AUSI31 210000 IHCL INCL 9.04 14.08 9.00 7.20 15.20 1.60 46.72
19 AU5041 250000 INCL INCL 9,04 14,00 9,00 7,20 15,20 1,60 46,72
19 AUSI41 250004) INCL INCL 9,04 14,4)8 9,00 7,20 15,20 1,60 46,72
20 AU5061 320000 INCL INCL 9,04 14,06 9,00 7,20 15,20 1,60 46,72
21 AU5061 370000 INCL INCL %04 14.06 9.00 7.20 15.20 1.60 46.72
22 A84 30000 INCL 3-15000 N/AV N/AV NIAV NIAV N/AV N/AV NIAV
23 AH 49950 INCL INCL HIAV H/AV NIAV HIAV HIAV NIAV N/AV
24 AS ENZYHE 65950 INCL INCL N/AV N/AV N/AV N/AV NIAV NIAV NIAV
25 I LIPJl) 59i0 INCL IHCL NIAV HIAV NIAV NIAV HIAV N/AV N/AV
26 HIH[ IDEAL 99500 INCL [NCL NIAV NIAV NIAV NIAV NIAV NIAV NIAV
27 AS IDEAL 120000 INCL INCL NIAV NIAV HIAV N/AV H/AV NIIW NIAV
26 CX3 39950 INCL 3-15000 NIAV NIAV NIAV NIAV NIAV HIAV N/AV
29 CX4 APP67500 3-15000 NIAV NIAV HIAV N/AV HIAV NIAV N/AV
30 CX5 APP72500 INCL 3-15000 NIAV N/AV N/AV NIAV N/AV N/AV N/AV
31 CX7 APP95000 INCL 3-15000 NIAV NIAV NIAV H/AV N/AV N/AV NIAV
32 ASCA 16000 N/A [NCL N/AV N/AV N/AV NIAV N/AV N/AV N/AV
CHEHILYDER 60000 6.00 NIDA 4.00 NIDA N/DA N/DA NIDA
34 6YSTEH4 3504)0 N/DA N/DA N/DA N/DA N/DA N/DA N/DA
35 PRINtA II 200000 NIAV YET INCL 7,35 24.04) 8,60 17,65 16.00 N/I)A 73.60
36 CORNINO 500 140000 INCL INCL 24,04) 26,00 16,00 25,00 12,04) NIi 94.
37 CORHINO 570 75000 INCL AP 15000 NIDA NIDA NII)A NII)A NIDA N/i)k N/DA
38 CORNINO EXPRESS 30000 N/A SY8 DEP NO UIE NIOA N/DA N/DA NIDA N/DA N/DA
39 CPA 30000 NIAV VARIABLE NO UIE 11,49 11.66 10.79 15,50 1.49 NO UIE
40 DACOB 2 65000 14800 VARIADLE 16,33 19,10 12,14 12,46 15,75 3,01 78,79
41 BIHOISION 380 50000 INCL 5000 14,91 60,00 30,04) 40,00 40,00 5,00 184,91
42 DINOIOH 760 90000 INCL INCL 14,91 80,00 30,00 40,00 40,00 5,00 144,91
43 OR[IIMR 6-400 60000 9000 14004) 12,90 17,36 4,59 9,98 22.77 0,95 62.81
44 ORKINER 6-450 68000 9000 14000 12,90 17,36 4,59 9,99 22,77 0,95 62,61
45 INOOI 2 130000 9000 INCL 12,90 17,36 4,59 9,98 22,77 0.95 62,81
46 TANOEH 3 180000 9000 INCL 12,90 17,36 4,59 9,96 22,77 0.95 62,61
47 MSI$ 21 47000 3000 5500 19,50 17,80 11,50 24,50 19,20 0,60 93,30
46 HON d)000 5000 5500 9,60 11,60 5,50 24,50 19,50 0,80 63,50
49 HONARCH PLUS 2 136000 1004)0 INCL 9,60 11,60 5,50 24,50 11,50 0,60 63,50
50 EKTACHEH DT 9100 INCL N/AP 216. 343,00 N/AV 195,00 195,00 H/DA 985,60
51 EKTACH[H 700 112000 INCL NIAPP 64,00 134,04) 64,00 70,00 70,00 14,00 436,00
52 PROOR8S 49500 5004) VARIES 7,0 5,32 2,96 2,10 7,92 0,77
53 6PECIFIC 31500 5000 VARIES 7.0 5.32 2,96 2,10 7,92 0.77 23.00
54 NOVA BIOHED 1/1 9545 VARIOUS NIDA NIAV *
55 NOVA DIOHED 4.4 17990 VARIOUS N/DA NIAV 'J
56 NOVA BlOIl:O 5*5 14745 VMIOUB NIDA HIAV *
57 CODAS FARA 51100 8500 VARIES 15,60 7,40 6,90 6,40 14,30 N/DA 50,60
50 CODAS HIRA 30704) 6500 VARIES 15,60 9,40 6,90 7,40 14,30 N/DA 53,60
ASSIST 16000 H/AV NIAV NIDA HIDA NIDA NIDA NIDA NIDA N/DA
60 CHEH ILd)000 INCL INCL 3,50 21.00 9,00 12,00 16,04) NIDA 61,50
61 RA-I000 40000 5000 6000 8,63 25,67 9,35 23,59 17,27 NIDA 44,91
62 RA-2X 904)00 INCL INCL. 6,63 25,67 9,35 23,59 17.27 HIDA 64,91
63 DA-500 25004) N/AV 10000 NO U/E 25.87 9.35 23.59 17.27 NIDA NO U/E
64 DA-XT 50000 8000 INCL 8.83 25,87 9.35 23.59 17.27 N/DA 84.91
65 SHAC II 160000 INCL INCl. 30,10 30,10 30,10 30,10 30,10 NIDA 30,10
66 MA 2000 22X)0 INCL INCL 30. tO 30, I0 30.10 30.10 30,10 NIDA 30, tO
67 DEH PROFILER 39950 N/AV ll. NO U/E NIAV NIAV NIAV NIAV NIAV NIAV
il ULTilOLAB NIDA NIDA N/DA NO UIE 16,00 5,00 9,00 16,0 N/DA NIDA
IITItLAD 200 13773 3800 NIDA NIDA HIDA HIDA NIDA NIDA NIDA N/DA
55
j. Fyffe et al. Review of large biochemistry analysers
NO JNT 10.4 U/E 10,4 LFT 10.4HflE 10.4CABO 10..4tIISC 10.4 N.L 10.SI 10.Sb
ABBOTT VPS N/DA
2 PARALLEL 0
3 PERSPECTIVE 3.5O
4 ENCOK II NO UIE
5 IRIT 2.15
6 HITACHI 704 1.25
HITACHI 705 1,25
8 HITACHI 737 1.25
9 EASY 0
10 [POB NO U/E
11 IRIS 10,00
12 [XgEL 10,00
13 EXTRA 10,00
14 AUSO21 0
15 AUSI21 0
16 AU5O31 0
17 AUSI31 0
18 AU5O41 0
19 AUSI41 0
2O AUS061 0
21 AU5O61 0
22 A84 0
23 ABO 0
24 A8 ENZYHE 0
25 AS LIPID 0
26 iIINI IDEAL 0
27 AS IDEAL 0
28 CX3 0
29 CX4 0
30 CX5 0
31 CX7 0
32 ABCA 2.00
33 CHERILYBER N/DA
3 SYEM N/SO
35 lISlIA II 1.50
36 CONIIJJ6 580 0.50
37 CORNIN6 570 N/OA
38 COIIINO EXPRESS NO U/E
39 CPA NO UIE
4O ,CO8 2
41 DIIIENOION 380 6.40
42 DINENOION 760 6.40
43 OREINER 6-400 1.96
44 6REIMt 6-450 1,96
45 TANDEII 2 1.96
46 TANDElt 3 1.96
47 6ENHIS 21 0
48 HONARCH 8.70
49 NONMCH PLUS 2 8.70
50 EKTACHEIt DT 0
51 EKTACHEH 700 0
52 PROSIIEgB
53 SPECIFIC 3.00
54 NOVA BIOHEll 14,1 N/AV
55 NOVA BIOIIE| 44,4 N/AV
56 NOVA BIOHED 54,5 N/AV
57 CODAS FARA 15.000
56 CODAS ItlRA 11.00
59 ASSIST N/DA
60 CHOI 1.24
61 RA-I000 9,05
62 Rk-2X 9.05
63 IIA-500 MO U/E
64 DA-IT
65 IIMC II 46,04
66 MA 2000 46.04
67 DEll PNOI:II.BI MIAV
M ULTROLM NOU/E
69 VITALA6 200 II/DA
N/DA N/OA M/DA NIDA MIDA NIDA MIDA
0 0 0 0 0 I.BN. 42P
4.90 2.60 2.10 2.10 72.09 1.2111. 42P
O.93 O.93 O.93 O.93 NO U/E O.25HL 9IP
2,15 2.15 2.15 2,15 2.15 I. IN. 9OP
1.25 1,25 1.25 1.25 1.25 O.301iL 98P
1,25 1.25 1,25 1.25 1,25 O,44HL 96P
1.25 1.25 1.25 1.25 1.25 O.22HL 96P
0 0 O 0 O IHL BOP
0 0 0 0 0 0.2N. 45P
17.50 I0.00 7,50 7,50 42,50 0.2N. 45P
0 0 0 0 I0.00 0.31tL 45P
17,50 10,00 7.50 7.50 42.50 O,3MIL 4
0 0 0 0 0 2N. 45P
0 0 0 0 0 2N. 45P
0 0 0 0 0 2N. 45P
0 0 0 0 0 45P
0 0 0 0 0 45P
0 0 0 0 0 2N. 45P
0 0 0 0 0 21tL 4
0 0 0 0 0 2N. 4
0 0 0 0 0 N/AV N/AV
0 0 0 0 0 N/AV N/AV
0 0 0 0 0 NIAV NIAV
0 0 0 0 0 N/AV N/AV
0 0 0 0 0 N/AV NIAV
0 0 0 0 0 NIAV NIAV
0 0 0 0 0 MIAV NIAV
0 0 0 0 0 NIAV NIAV
0 0 0 0 0 WAY NIAV
0 0 0 0 0 NIAV N/AV
2,00 2,00 2,00 2,00 2,00 VARIES VARIES
N/DA NIDA MIDA NIDA NIDA NIDA N/DA
NI NIDA NIDA NIDA N/DA NIDA NIDA
1.50 1.50 1.50 1.50 1.5O 2N. DP
0.50 0.50 0.50 0.50 0.5O 5N. lOOP
HIDA M/DA H/DA M/DA N/DA 5N. lOOP
2.00 2.00 2.00 2.00 2.00 IN. lOOP
N/DA MIDA HIDA NIDA MIOA O. 121tL 2P
M/DA MIDA NIDA NIDA M/DA 0,9211L 22P,
6.40 6.40 6,40 6.40 6.40 0,74N. AP27OP
6.40 6040 6.40 6.40 6.40 O.74N. AP27OP
1.96 1.52 0.86 0.66 5.92 (1 IIL VARIES
1.96 1.52 0.86 0.66 5.92 (1 Hi. VARIES
1.96 1.52 0.86 0.06 5.92 <2 N. VMIES
1.96 1.52 0.86 0.66 5.92 (2 IlL VARIES
0 0 0 0 0 0.70ILL 2OP
20.30 11.60 B.70 9.70 58.00 O.301tL 20P
20.30 11.60 8.70 6.70 58.00 0.3L 2OP
0 0 0 0 0 1.3N. IIIP
0 0 0 0 0 O,95HL 125P
4.00 6.00 6.O0 6.O0 35,00 NIDA NIDA
14.00 8.00 6.00 6.00 35.00 NIDA NIDA
INCL INCL
e INCL INCL
J INCL INCL
25.5O 15.00 11.50 15,00 62,00 0.3N. VARIES
18.50 11.00 6.50 11.00 60.00 0.3ON. VMIE$
N/DA NIDA N/DA MIDA MIDA NIDA N/DA
2,30 1.15 1.24 1,24 7,17 HIDA H/DA
16.64 9.05 7.16 7.16 49.06 1.2 146Y 85P
16.64 9.05 7,16 7.16 49.06 1.2 14DY 85P
16.64 9,05 7.16 7,16 NO U/E 1,2 140Y OSP
16,64 9.05 7,16 7,16 49,06 1.2 14DY 85P
46.04 46.04 46.04 46.04 46.04 MIDA HIDA
46.04 46,04 46.04 46.04 46.04 NIDA NIDA
NIAV NIAV NIAV NIAV N/AV NIAV NIAV
3,00 3.00 3,00 3.00 NIDA NIDA MIDA
N/DA MIDA MIDA NIDA N/DA l.OlIL N/DA
10.5c
M/DA
ION. 24HR
IOHL 24HR
IOHL IODAYS
ION.IODY/SHt.ID
3HI. 2DAYS
3HL 2DAYS
3N. 2DAYS
IN. IlK
5N. IlK
5N. INK
5N. IlK
5HL IlK
5N. IlK
5HL IlK
5N. IlK
5N. IlK
NIAV
N/AV
N/AV
N/AV
N/AV
N/AV
N/AV
NIAV
NIAV
N/AV
NIDA
PENDS ON CAL
ION. 2DAYS
ION. 2DAYS
IN. 2DAYS
3N. 24HR
31tL 24HR
3JIHL 6HRS
3IN. 6HRS
VMIES
VARIES
VARIES
VARIES
511L 7DAYS
5ill 7DAYS
511L 7DAYS
3N. 1DAY
91L 1DAY
NIDA
NIDA
INCL
INCL
INCL
NO REC CALl8
NO REC CALl6
NIDA
3N. 48HR
35L 48HR
48M
NIDA
N/DA
NIAV
5N.
56
Jo Fyffe et al. Review of large biochemistry analysers
NO INST NaC Nail Na C K N BICC DICN DICB CI CI N CI UREC UREN UREB CREC CREN CREB
ADDOTT VPS N/AV HIAV N/AV NIAV 7,6 2.9 3,4 271
2 PARALLEL 143 0,9 1,7 4,7 t,2 2,0 N/DA N/DA 10,0 1,3 2,9 340 1,5
3 PERSPECTIVE 143 0,8 1.7 4.7 1.2 2.0 N/DA N/DA 10,0 1,3 2,9 340
ENCORE lI N/AV N/A9 N/AV N/DA 6,7 2,0 3,5 106 2,9 4,1
5 SPIRIT 145 0,5 NIDA 4,1 1,2 N/DA 28,6 3,1 6,6 112 1,3 3,0 5,0 1,3 2,2 159 2,6 6,1
6 HITACHI 704 141 0,6 0,9 5,0 0,7 1,0 N/DA 102 0,6 1,1 6,3 1,1 1.7 300 1,4
HITACHI 705 144 1.1 0.9 4.3 1.2 1.3 N/DA N/DA 26.0 ].0 ].3 336 1.5 2.1
9 HITACHI 737 145 0,6 0,7 5,0 0.7 1,0 N/DA 102 0.6 1,1 15,5 1.2 1,6 404 0,9 1,4
9 EASY NIDA N/DA N/DA N/DA N/DA N/DA
10 EPOS N/AV N/AV N/AV N/AV 6,9 1.1 N/DA 119 0,5 N/DA
11 ERIS 177 0,9 H/DA 4.5 0,9 N/DA 16.0 2.1 N/DA 106 0,5 N/DA 6.0 1,1 N/DA 107 1,1 N/DA
12 EXSEL 177 0,9 N/DA 4,5 0,9 N/DA 18,0 2,1 N/DA 106 0,5 N/DA 8,0 1,1 N/DA 107 1,1 N/DA
13 EXTRA 117 0.9 N/DA 4.5 0.9 N/DA 18.0 2.1 N/DA 106 0.5 N/DA 8.0 1.1 N/DA 107 1.1 N/DA
14 AU 5021 N/DA N/DA N/DA N/DA N/DA N/DA
15 AU 5121 N/DA N/DA N/DA N/DA N/DA N/DA
15 AU 5031 N/DA N/DA N/DA N/DA N/DA N/DA
i7. AU 5131 H/DA N/DA N/DA N/DA N/DA H/DA
18 AU 5041 N/DA N/DA N/DA N/DA N/DA N/DA
19 AU 5141 N/DA N/DA N/DA N/DA N/DA N/DA
20 AU 5061 N/DA N/DA N/DA N/DA N/DA N/DA
2! AU 5061 NIDA N/DA NIDA N/DA N/DA N/DA
22 AS4 156 0,5 0,5 5.5 1.9 1,5 27.0 3,7 3,6 111 1,4 0,5 15.9 1.5 2.5 327 2,7 1.9
23 AS8 156 0.5 0,5 5.5 1,6 1,5 27.0 3.7 3.5 111 1.4 0.5 15.9 1.5 2.5 327 2.7 1.9
24 A8 ENZYHE 156 0.5 0.5 5.5 1.8 i,5 27.0 3.7 3.5 111 1,4 0.5 i5.9 1.5 2,5 327 2.7 1.9
25 AS LIPID 155 0,5 0.5 5.5 1,6 1.5 27.0 3.7 3.5 111 1,4 0,5 15,0 1.5 2,5 327 2.7 1,?
26 AS HINI IDEAL 156 0,6 0.6 5.5 1,8 1,6 27.0 3,7 3,6 111 1.4 0,5 16.9 1,5 2,6 327 2.7 1,9
27 AS IDEAL 156 0.6 0.6 5.5 l.O i.6 27.0 3.7 3.6 111 1.4 0.5 16.6 1.5 2.6 327 2.7 1.9
28 CX3 N/DA N/DA N/DA N/DA N/DA N/DA
29 CX4 N/DA NIDA N/DA N/DA N/DA N/DA
30 CI5 N/DA NIDA N/DA H/A H/DA N/DA
31 CX7 N/DA N/DA N/DA N/DA N/DA H/DA
32 ASCA N/AV N/AV N/DA N/DA N/DA N/DA
33 CHEHILYSER 139 l.O 1,2 4,9 0,8 l,O 27,0 1,6 2.2 N/DA 21.5 0.6 1.9 375 1.5 1,6
34 SYSTEH4 135 0,9 0,9 4,7 0,6 0,9 9,0 2,1 2,3 N/DA 8.8 1.7 1,? 143 0,? 1,0
35 PRIRHA II 140 <1,0 i,O 4,0 <t,O i.O 20,0 2,0 3.0 tO0 1,0 2,0 7,0 1,0 2,0 60 2.0 ,0
36 CORNIN6 580 138 0,5 0,6 4,1 0,7 0.5 N/DA tOt 0,4 0,5 4.0 3,1 1.4 237 1,0 1,0
37 CORNIN6 570 N/DA NIDA N/DA N/DA NIDA 2.0 3.0 N/DA 2.5 3.0
39 CORNIN6 EXPRESSNIAV N/AV N/DA N/DA NtDA N/DA
39 CPA N/AV N/AV N/DA 3.0 N/DA N/DA 2.0 4.0 N/DA 2.5 4.0 N/DA 2.0 3.5
40 DACOS 140 0.4 1.5 4.0 0.4 2,0 22.0 2.3 4.5 99 0.5 2.5 5.9 1.4 2.5 112 2,7 3.7
41 DIHENBION 390 151 i,7 2,5 4.3 2,0 2,3 N/DA 106 l.i 1.9 2,2 2,5 4,8 236 i.8 3.4
42 DIHENSIOH 760 151 1,7 2,5 4,3 2,0 2,3 N/DA 106 1,1 1,9 2,2 2,6 4,9 236 1,6 3,4
43 6REINER 6-400 136 0.5 0,6 4,3 1.0 1,5 27,3 3.i NIDA 101 0.9 1.5 16.5 0,9 1.7 345 0.4 1.4
44 6REINER 6-450 136 0,5 0,8 4,3 1,0 1,6 27,3 3,1 N/DA 101 0.9 1,5 16,5 0,9 1,7 345 0,4 1,4
45 TANDEH 138 0,5 0,6 4,3 i,O 1,6 27,3 3,1 N/DA 101 0,9 1,5 16,5 0,9 1,7 345 0,4 1,4
45 TANDEH 139 0,5 0,9 4,3 1,0 1,6 27,3 3,1 N/DA I0i 0,9 1.5 16,5 0,9 1,7 345 0,4 1,4
47 6ENESlS 21 141 0,5 1,1 4,1 1,3 1,3 27,0 2,4 5,2 95 1,0 1,5 15,5 2.1 2,5 88 2,2 4.4
48 HONARCH 139 0,5 1,2 4,3 0,2 0,9 25,0 1,6 5,9 99 0,4 I,I 18,2 1,4 2,9 102 2,0 3,3
49 HONARCH PLUS 139 0.5 i.2 4.3 0.7 0.9 25.0 1.5 5.9 99 0.4 1.i 18.2 i.4 2.9 102 2.0 3.3
50 EKTACNEH DT 127 1,1 1.1 5,9 1.4 1.5 23.0 4,8 4.9 108 i.O 1.5 7.8 2.4 2.5 195 5,3 5,7
5i EKTACHEN 700 129 0,7 1,1 4.4 1.4 1.5 24.1 4.2 4.2 101 0.7 0.9 12.3 1.9 2.8 521 1.0 1.7
52 PROORESS 140 0,4 0,6 4,5 1,4 1,3 N/DA N/DA 6,1 1,3 2,5 120 1,5 3,0
53 SPECIFIC N/DA N/DA NIDA N/DA N/DA NIDA
54 NOVA l/l 150 (0,7 (1,5 150 (0,7 (1,5 N/AV N/AV N/AV N/AV
55 NOVA 4+4 i50 10.7 (1.5 150 (0,7 (1.5 22,0 0,8 1,6 175 (0,7 (1,5 N/AV N/AV
55 NOVA 5/5 150 (0.7 (1,5 150 (0,7 (1.5 N/AV i60 <0,7 <1,5 N/AV N/AV
57 CODAS FARA 140 0.5 N/DA 5,0 0,5 N/DA N/DA N/DA 4,9 1.8 2, i4i i,9 1,3
50 CODAS HIRA 140 0.5 N/DA 5,0 0,5 N/DA N/DA N/DA N/DA N/DA
59 ASSIST N/DA N/DA H/DA N/DA N/DA N/DA
60 CHEH 142 0,6 N/DA 4.6 1,0 N/DA 26,0 2,0 N/DA N/DA 7,1 N/DA 1,7 N/DA
61 RA-IO00 130 0,6 N/DA 4,5 l,i N/DA 25,0 2,4 N/DA 103 0,7 N/DA 13,0 i,3 N/DA 452 0,5 N/DA
62 RA-2X 130 0,5 N/DA 4,5 1,1 N/DA 25,0 2,4 N/DA 103 0,7 N/DA 13,0 1,3 N/DA 452 0,5 N/DA
63 RA 500 N/DA N/DA N/DA N/DA N/DA N/DA
64 RA-XT N/DA N/DA N/DA N/DA NIOA N/DA
65 IAC II |49 0.6 N/DA 8.9 1.3 N/OA 23.0 1.5 N/DA 104 0.9 N/DA 10.5 1.2 N/DA 340 1.6 N/DA
66 8RA 2000 149 0,5 N/DA 6,9 1,3 N/DA 23,0 1,5 N/DA 104 0,9 N/DA 10,5 1,2 N/DA 340 1,6 N/DA
67 6EH-PROFILER N/DA NIDA N/DA N/DA NIDA N/DA
60 ULTROLAD N/AV N/AV N/AV N/DA N/DA N/DA
69 VITALAD 200 N/AV N/AV N/DA N/OA N/DA H/DA
57
j. Fyffe et al. Review of large biochemistry analysers
NO INST TP C TP N TP B ALBC ALBN ALBB BILC BILN BILB ALPC ALPN ALPB HBDC HBI)N HBDB ASTC ASTN ASTB
ABBOTT VPS 68 1,0 0,8 42 0,8 0,? 84 1,6 2,3 218 1,7 3,8 24? 1,3 2,1 IIO 1.3 2.9
PARALLEL 60 ],0 2,0 45 ],5 3,0 70 [,0 [,7 [50 2,0 4,4 N/AV 80 2,5 4.9
PERSPECTIVE 60 1,0 2,0 45 1,5 3,0 70 I,O 1,7 I50 2.0 4,4 N/AV 80 2,5
4 ENCORE II 60 1.2 2.7 46 1.2 2.4 104 1.1 2.5 157 1.0 2.1 170 1.2 2.1 83 1.2 4.5
5 SPIRIT 53 1,3 3,7 37 1.1 2,7 15 3,3 5,0 98 I,I 4,5 N/AV 43 2,4
6 HITACH] 704 55 0,8 I,O 36 1.0 1,2 39 0,5 1,3 490 0,8 1,5 N/DA 100 0.7
HITACHI 705 60 1,9 3,0 37 1,1 t.5 7,1 0,6 1,4 1235 0,6 1,4 250 1,2 2.2 471 1,0 2,0
B HITACHI 737 63 0.8 1.2 42 I.t 2.1 74 0.8 1.3 504 0.8 1.1 N/BA 105 0.7 1.1
? EASY N/DA N/DA NIDA N/DA N/DA N/DA
lO EPOS 44 I.O N/DA 2! O.B N/OA 20 2.7 N/DA 257 1.4 NIBA 69 1.2 N/DA 27 1.6 N/DA
I! ERZS 69 O.B N/DA 26 I.! N/DA 20 1,1 N/DA 286 0.7 N/DA 233 1.2 N/DA 44 1,1 N/DA
12 EXSEL 69 0.8 N/DA 26 l.l N/DA 20 [.! N/DA 286 0.7 N/DA 233 1.2 N/DA 44 l.l N/DA
13 EXTRA 69 O,S N/DA 26 1,1 N/DA 20 1,1 N/DA 286 0.7 N/DA 233 1.2 N/DA 44 1,1 N/DA
14 AU 5021 N/DA N/DA N/DA N/DA N/DA
15 AU 5121 N/DA N/DA N/DA N/DA N/DA
[6 AU 503! N/DA N/DA N/DA N/DA NIDA
17 AU 5131 N/DA N/DA N/OA N/DA N/DA
18 AU 5041 N/DA N/DA N/DA N/DA N/DA
19 AU 5141 N/DA NIDA N/DA N/DA N/DA
20 AU 506! N/DA N/Ok N/DA N/DA N/DA
2! AU 508! N/DA N/DA N/DA N/DA N/DA
22 AS4 7! 2.4 2.9 48 0.7 !.2 84 3.4 7.0 N/AV N/AV
NIDA
N/OA
N/DA
NIDA
NIDA
N/DA
N/DA
N/DA
N/AV
97 3.0 2,7
97 3.0 2.7
97 3.0 2.7
97 3.0 2.7
97 3.0 2.7
N/DA
N/DA
N/BA
N/DA
N/DA
N/AV
N/AV
23 AS8 71 2,4 2,9 49 0,7 1.2 84 3.4 7,0 146 1,4 2.5 N/AV
24 AS ENZYH 7! 2,4 2,9 40 0,7 !,2 64 3,4 7,0 146 1,4 2,5 N/AV
25 AS LIPID 71 2.4 2.9 48 0.7 1,2 84 3.4 7.0 146 1,4 2.5 N/AV
26 AS HINI IDEAL 71 2.4 2.9 48 0.7 1,2 94 3,4 7,0 146 1,4 2.5 N/AV
27 AS IDEAL 71 2.4 2.9 48 0.7 1.2 04 3.4 7.0 146 1.4 2,5 N/AV
26 CX3 N/DA N/DA N/DA N/DA N/DA
29 CX4 N/DA N/DA N/DA N/DA N/DA
30 C|5 N/DA N/DA N/DA N/DA N/DA
31 CI7 N/DA N/DA N/DA NIDA N/DA
32 ASCA NIDA N/DA N/DA N/DA N/DA
33 CHEHILYSER 65 I.O 1.2 32 !.3 1.5 23 0.9 1,3 175 1.0 1.4 N/AV
34 SYSTEH4 72 O.O 1.0 32 I,O 2.1 21 1.1 1.4 196 2,0 2,2 N/AV
5 PRIBRA II 60 (t,O l.O 30 (1.0 2,0 30 1,0 3,0 190 2,0 4,0 150 2,0 4,0 40 2,0
36 CORN!N6 560 46 0,7 0,6 30 1,6 i,8 %1 1,2 i,2 73 2,4 2,9 N/DA 22 5,5 i,9
37 CORNIN6 570 N/DA N/DA N/DA N/DA N/DA N/DA
39 CORNIN6 EXPRESSN/DA N/DA N/DA N/DA N/DA N/DA
39 CPA N/DA N/DA N/DA N/DA N/DA N/DA
40 DAC00 2 62 0.7 1.4 36 1.1 1.5 35 2.8 4.3 84 1.8 3.6 N/DA 70 2.1 3.3
41 DIHENSION 360 67 1,5 2,0 37 2,9 2,9 32 0,9 3,4 N/DA N/AV 166 3,0 4,4
42 DIHENS]ON 760 67 1.5 2.0 37 2.9 2,9 32 0,9 3,4 N/DA N/AV 166 3,0 4,4
43 6RE[NER 9-400 71 0.5 1,5 39 0,6 1,8 93 0,4 1,4 217 1,I 2,4 561 2.6 3,2 149 1,5 2.5
44 6RE]NER 6-450 71 0,5 1,5 39 0,6 1,0 03 0,4 1.4 217 1.1 2.4 581 2,6 3,2 149 !,5 2,5
45 TANDEH 71 0,5 1,5 39 0.6 1,8 83 0,4 1.4 217 1,1 2,4 516 2,6 3,2 149 1,5 2.5
46 TANDEH 3 71 0.5 1,5 39 0,6 1.8 03 0.4 1,4 217 1,1 2.4 501 2.6 3.2 149 1.5 2.5
47 6ENEO[O 21 60 0.8 1.7 38 1,I 24 5 1.3 2.! 126 1.7 3,5 400 1.6 2.1 90 0.7
48 HONARCH 66 1.2 2,5 39 0.6 3.0 65 1.5 2.5 74 1.3 3.3 370 1.6 4.3 75 1.1 2.4
49 HONARCH PLUS 68 1,2 2.5 38 0.6 3.0 65 1,5 2,5 74 1,3 3.3 370 1.,6 4.3 75 1.1 2.4
50 EKTACHEH DT 66 2.2 2.2 N/AV 33 5.3 7,4 74 2,5 4,6 N/AV 70 5,6 7.1
51 EKTACHEH 700 72 1.8 2.2 36 1.7 1.7 69 2.0 3.2 99 2.5 4.2 N/AV 202 1.2 2,8
52 PRO6REO$ 70 0.6 I.O 34 1.0 1.3 26 2.2 1.6 230 2.2 2.8 N/DA 63 1.2 2.2
53 SPEC[F[C N/DA N/DA N/DA NIDA N/DA N/DA
54 NOVA 1+I N/AV N/AV N/AV N/AV N/AV N/AV
55 NOVA 4+4 N/AV N/AV N/AV N/AV N/AV N/AV
5 NOVA 5+5 N/AV N/AV N/AV N/AV N/AV N/AV
57 CODAO FARA 56 O.O 1.2 62 1.6 1.8 34 0.9 2.9 114 0.4 1.2 263 1.0 1.7 169 1.7 2.1
56 CODAE N]RA 72 1,2 1,7 36 1.6 2.5 112 1,0 1,5 192 0.6 0.9 274 1,9 3.1 95 0,7 1.1
N/OA
163 N/DA 1.7
300 1.3 N/DA
300 1,3 N/DA
H/DA
N/OA
200 1,3 NIDA
200 1,3 N/DA
N/DA
N/DA
NIDA
59 ASSIST NIDA N/DA N/DA N/DA N/DA
60 CHEH N/DA 35 N/DA 1.4 NIDA 162 NIDA 1,6 N/AV
61 RA-IO00 75 1,0 N/DA 43 1,3 N/DA 95 2,0 N/DA 64 0,7 N/OA N/AV
62 RA-2X 75 1,0 N/DA 43 1,3 N/DA 05 2,0 H/DA 64 0,7 N/DA N/AV
63 RA-500 NIDA N/DA N/DA N/DA N/DA
64 RA-XT N/OA N/DA H/DA N/DA N/DA
65 8HAC [l 64 l,I N/DA 36 1.3 N/DA 76 1.5 NIDA 153 1,3 N/DA NIAV
DRA 2000 64 1.1 NIDA 38 1.3 N/DA 76 1.5 N/DA 153 1.3 N/OA NIAV
67 9EH-PROFILER N/DA N/DA N/DA N/DA N/DA
6 ULTRoLAD N/DA N/OA N/DA NIOA HIDA
69 VITALAD 200 NIDA N/DA N/DA H/DA N/DA
58
J. Fyffe et al. Review of large biochemistry analysers
NO INST ALTC ALTN ALTB 66TC 66Til 66TB LI)HC LDHtl LI)HB CK C CK it CK B CALC CALII CALB P04C P04tt P04B
ABBOTT VPS 45 1.8 2.3 73 1.0 3.1 310 1.7 2.5 64 2.4 4.0 2.25 1.5 1.0 1.78 1.3 2.1
2 PARALLEL 60 ;S,O 6,0 40 3.0 8,0 N/AV ???? 2.8 5,5 2,50 1,0 1,5 1.30 1,0 1.6
PERSPECTIVE BO 3.0 6.0 40 3.0 6.0 N/AV N/I)A 2.50 l.O 1.5 1.30 1.0 1.6
ENCORE II 76 0,9 5.2 89 1.5 6,0 230 1.2 2.1 83 1. 3,8 2.75 1,3 3,4 2.97 1,3 3,8
5 SPIRIT 30 2.4 5.:S N/DA 370 1.7 4.9 146 1.8 5.5 2.35 2.0 4.5 2.20 1.4 3.9
HITACHI 704 55 1,3 2,2 104 1,0 1.3 329 1,1 1,9 360 0,7 1,5 2,30 0.9 1,4 N/DA
HITACHI 705 513 0.5 1.6 840 0.6 1.3 N/DA 71 1.0N/I)A 4.07 1.5 2.4 1.77 2.3 1.9
6 HITACHI 737 128 0,7 1,1 176 0,9 1,2 498 1,2 1,5 365 0,7 1,5 2,63 0.9 1.9 1,50 1,2 1.7
N/i)A N/i)A N/BA 46 N/I)A 3.0 N/BA N/I)A
30 1.9 N/i)A 32 1.5 N/DA 146 1,3 N/DA 46 2,0 N/DA 1.33 0.7 N/DA 1.64 2.3 N/DA
44 1,2 NIDA 62 0,5 N/i)A 415 1,4 N/BA 48 2,0 NI])A 2,59 l,l N/DA 1,12 1,8 H/DA
44 1,2 N/DA 62 0,5 N/DA 415 1,4 N/I)A 48 2.0 N/DA 2,59 l.l N/DA 1,12 1,8 N/I)A
44 1,2 N/DA 62 0,5 H/I)A 415 1,4 H/DA 48 2,0 N/DA 2,59 1,1 N/I)A 1,12 1.8 H/I)A
N/I)A N/DA N/DA N/I)A N/I)A N/I)A
N/DA N/DA N/DA N/I)A N/I)A
N/DA N/DA N/DA N/I)A N/DA N/DA
H/I)A N/I)A N/I)A N/DA N/DA
N/DA N/DA N/DA N/I)A H/DA N/OA
N/I)A N/DA N/DA N/DA N/DA
N/DA N/I)A N/DA N/DA N/DA N/I)A
N/I)A H/DA H/OA N/DA H/BA H/I)A
N/AV N/AV N/AV N/AV 3.30 1,3 1,5 1.94 1,5 N/DA
9 EASY
10 EPOS
11 ERI6
12 EXSEL
13 EXTRA
14 AU 5021
15 AU 5121
16 AU 5031
17 AU 5131
18 AU 5041
19 AU 5141
20 AU 5061
21 AU 5081
22 AS4
23 ASS
24 AS ENZYHE
25 AS LIPID
95 3,2 3.7 110 2.7 3.9 306 1.6 27 148 2.0 2.3 3,30 1,3 1.5 1.94 I.SN/DA
95 3.2 3,7 lLO 2,7 3,9 306 1,6 2.7 146 2,0 2.3 3,30 1.3 1,5 1.94 I,SN/I)A
95 3.2 3.7 110 2.7 3.9 306 1.6 2.7 148 2.0 2,3 3.30 1,3 1.5 1,94 I.SN/DA
26 AS HINI II)EAL 95 3,2 3,7 I10 2,7 3,9 306 1.6 2,7 148 2,0 2.3 3.30 1,3 1.5 1,94 1,5 N/DA
27 AS II)EAL 95 3,2 3.7 110 2,7 3.9 306 1.6 2,7 146 2,0 2,3 3.30 1,3 1,5 1,94 1.5 N/DA
28 CX3 NtDA N/DA N/DA H/DA N/DA N/DA
29 CX4 N/DA N/DA N/DA N/I)A NII)A N/DA
30 CX5 NIBA H/I)A N/I)A N/I)A N/OA H/I)A
31 CX7 H/DA N/DA N/I)A N/DA N/DA
32 ASCA N/DA N/DA N/DA N/DA N/DA N/DA
33 CHEHILYSR H/AV 56 1,5 1,5 N/AV H/BA 2,40 1,3 1,5 1,05 0,9 1,0
34 SYSTEH4 N/AV 56 1.3 1.6 N/AV N/I)A 3,02 0,9 1,1 1,02 0.9 1,1
35 PRISHA II 40 2.0 5.0 60 2,0 5,0 300 2.0 4,0 150 2,5 3.0 2.00 (1,0 2.0 1.00 1,0 3.0
36 560 ALLIANCE 19 9,6 5.1 19 4,9 2.4 120 3.2 1,4 135 1.5 1,6 1,86 0.7 1.1 N/DA l.O 2.0
37 500 N/DA N/DA N/DA N/DA N/DA N/DA
38 CORNIN6 EXPRESS N/DA N/DA H/I)A N/I)A N/I)A
39 CPA N/OA N/DA N/I)A NIDA N/A NIDA
40 DACOS 108 1.6 4.6 152 1.1 2.3 682 0.8 1.0 177 1,2 2.0 2.60 0.9 2.4 1.26 0.8 1.7
41 I)ZHENGION 390 68 3.1 4.9 28 5.0 6.9 355 1.4 3.3 100 1.4 1.7 N/DA 1.21 1.3 3.6
42 I)IHEHSION 760 69 3.1 4,9 28 5,0 6.9 355 1.4 3,3 100 1,4 1,7 NIDA 1,21 1,3
43 6-400 59 2.8 4.9 140 0.9 1.6 240 1.5 2.0 275 1.0 5.1 2.62 0,3 1.7 1.03 0.8 2.4
44 6-450 59 2.9 4,9 140 0.9 1,6 240 1.5 2.0 275 1.0 5.1 2.62 0,3 1,7 1,03 0.8 2,4
45 TANDEH 2 59 2.6 4.9 140 0,9 1,6 240 1.5 2,0 275 1.0 5.1 2.62 0,3 1,7 1,03 0,6 2,4
46 TANDEH 3 59 2,B 4.9 140 0.9 1.6 240 1.5 2.0 275 1.0 5.1 2.62 0.3 1.7 1.03 0.8 2.4
47 8ENESIS 21 89 0.7 1.3 63 1.3 2.9 460 1.6 2.1 94 1.7 3.4 2.24 0.9 2.0 0.65 0.9 1.6
49 NOHARCH 76 1,2 2,3 69 0,9 2,5 380 1,7 4,2 121 1,3 3,1 2,42 1,4 1.7 1.17 1.4 2,0
49 HONARCH PLUS 2 76 1.2 2,3 69 0,9 2,5 380 1.7 4.2 121 1,3 3,1 2,42 1,4 1,7 1,17 1,4 2,0
50 EKTACHEH DT 71 4,3 4,6 90 2,2 2,3 649 0,9 2,9 175 1,8 3,0 N/AV N/AV
51 EKTACHEH 700 164 1,0 2.8 89 1,7 2,7 489 2,6 3.2 109 4,1 8,2 2,35 0,9 1,3 1,06 2,1 2,1
52 PROBRESS 40 1,5 3,0 50 1,8 2,4 81 2,7
SPECIFIC N/DA N/I)A N/I)A N/DA
54 NOVA 1+1 N/AV N/AV N/AV N/AV
55 NOVA 4+4 N/AV H/AV NIAV HIAV
56 NOVA 5+5 N/AV N/AV N/AV N/AV
57 CODAS FARA 142 1,6 2,1 77 0,9 1,6 440 1.2 1,7 H/DA
58 CODAS HIRA 97 0,5 0,6 92 0,9 1,5 271 1,9
59 ASSIST N/DA N/DA N/OA H/DA
60 CHEH NIDA N/DA 130 N/DA 1,6 N/I)A
61 RA-tO00 300 NIOA 1.3 106 NIDA 1.0 H/AV HIDA
62 RA-2X 300 N/I)A 1.3 108 N/DA l,O N/AV N/DA
63 RA-500 N/DA N/DA N/DA H/DA
64 RA-XT HIDA N/DA H/I)A NIDA
65 8HAC II 38 3.9 H/DA 65 4,3 N/DA 422 2.3 NIDA N/DA
66 SRA 2000 6 3,6 NIDA 65 4,3 N/DA 422 2,3 N/DA N/DA
67 6EH-PROFILER HIA NIDA HIDA NIDA
68 ULTRoLAD N/DA N/DA N/DA N/DA
69 VITALAD 200 N/DA N/Ok N/DA N/DA
2,26 1.2 1.1 1.20 1,2 2,0
N/DA N/DA
N/AV N/AV
N/AV N/AV
N/AV N/AV
2,49 0,6 1.4 2,50 1.2 2,9
2,50 0,9 1,5 2,40 2.0
H/I)A H/DA
N/DA N/DA
2.25 N/DA 1.2 N/AV
2,25 N/I)A 1,2 N/AV
N/DA H/DA
N/I)A
2,60 0,9 N/DA 1,40 1,4 N/DA
2,60 0,9 N/I)A [,40 1,4 N/DA
N/DA NIDA
N/DA
N/OA N/DA
59
j. Fyffe et al. Review of large biochemistry analysers
NO INST CHOC CHOM CHOB T6 C T6 II T6 B URAC URAtl URAB 6LUC 6LUll 6LUB
ABBOTT VPS 2.3 1.3 2.7 0.76 1.1 4.5 654 0.6 0.6 13.8 3.2 4.4
PARALLEL 4,0 1.5 2.6 N/AV 370 1.5 3.0 ???? 1.0 3.5
PERSPECTIVE 4.0 1.5 2.6 ???? 2.5 4.1 370 1.5 3.0 ???? 1.0 3.5
ENCORE II 10.2 1.2 3.8 3.69 0.8 4.2 570 I,S 1.9 15,6 0.6 2.4
5 SPIRIT 3.3 1.0 3,2 N/DA 360 1,2 N/DA 15.8 1.0 2.3
6 HITACHI 704 2.8 1.0 2.0 0.85 0.6 1.5 280 0.7 1,6 N/DA
HITACHI 705 8.2 0.5 1.6 3.44 1.1 l.B 539 0.6 1.? 5.6 O.6N/I)A
8 HITACHI 737 4.6 0.9 1.1 1,79 1.0 1.2 625 1,0 1.4 8.3 1.3 1.2
N/DA N/I)A N/DA 10.0 N/DA 1.5
5,0 0,5 N/DA 0,86 2.6 N/DA 455 0,8 N/DA 10.0 1,0 N/DA
2.4 0.6 N/DA 1.50 0.9 N/DA 458 1.2 N/I)A 10.0 0.9 N/I)A
2.4 0.6 N/DA 1.50 0.9 N/DA 458 1.2 N/DA 10.0 1.0 N/DA
2.4 0.6 N/I)A 1.50 0.9 N/i)A 458 1.2 N/I)A 10.0 0.9 N/I)A
? EASY
10 EPOS
12 EXSEL
13 EXTRA
14 AU 5021
]5 AU 5121
16 AU 5031
17 AU 5131
18 AU 5041
19 AU 5141
20 AU 5061
21 AU 5081
22 A84
23 AS8
24 AS ENZYHE
25 AS
N/I)A N/DA N/DA N/I)A
N/DA N/OA N/I)A N/DA
N/DA NIDA N/DA N/DA
N/I)A N/I)A N/I)A N/I)A
N/I)A N/DA N/DA N/DA
N/DA N/I)A N/I)A N/I)A
N/DA N/I)A N/DA N/I)A
N/DA N/I)A N/I)A N/DA
N/AV N/AV N/AV 4.3 1.2 1.8
27 AS II)EAL
28 CX3
29 CX4
30 CX5
31 CX7
32 ASCA
33 CHEHILYSER
34 SYSTEH4
5 PRISHA II
5.0 2.6 N/I)A 1.90 3.0N/DA 510 1.9 N/I)A 4.3 1.2 l.B
5.0 2.6 N/DA 1.?0 3,0 N/DA 510 1.9 N/DA 4.3 1,2 1.8
5.0 2.6 N/I)A 1.90 3.0 N/I)A 510 1.9 N/DA 4.3 1.2 1,8
26 AS HINI II)EAL 5.0 2.6 N/DA 1,90 3,0 N/DA 510 1.9 N/DA 4.3 1,2 1,8
5.0 2.6 N/i)A 1.90 3.0 N/DA 510 1.9 N/DA 4.3 1.2 1.8
N/DA N/DA N/DA N/DA
N/I)A N/I)A N/DA N/DA
N/DA N/DA N/DA N/DA
N/I)A N/I)A N/DA N/I)A
N/DA N/DA N/DA N/I)A
2.2 1.1 1.4 N/AV 460 2.0 2.3 N/I)A
1,9 1,2 1.5 NIAV 440 2,1 2.2 10.9 0,5 N/I)A
4.0 (1.0 1.0 1.00 (1.0 3;.0 400 1.0 2.0 5.5 1.5 2.0
3;6 CORNIN6 580 3.0 1.5 1.2 0.65 1.5 4.3 237 0.7 1,1 4.3 0.7 1,7
37 CORNJN6 570 N/DA N/DA N/DA N/i)A
36 CORNIN6 EXPRESS N/DA N/DA N/DA N/DA
39 CPA N/DA N/DA N/DA N/DA
40 DACOB 2 4.9 1.0 1.? 0.77 1.0 1.9 355 0.8 2.5 8.3; 0.8 1.3
41 I)IHENSION 3;80 NIDA N/DA 247 3;.0 4.3; 5.1 2.0 3;.4
42 DIHENSION 760 NIDA H/DA 247 3.0 4.3 5.1 2.0 3.4
43 6-400 4.7 1.3; 2.9 2.3;2 1.7 1.7 420 0.7 3.5 12.0 1.4 1.7
44 6-450 4.7 1.3 2.9 2.32 1.7 1.7 420 0.7 3.5 t2.9 1.4 t.7
45 TAHDEH 4.7 1.3 2.9 2.32 1.7 1.7 420 0.7 3;.5 12.6 1.4 1.7
46 TAHDEH 4.7 1.3 2.9 2.3;2 1.7 1.7 420 0.7 3.5 t2.9 1.4 1.7
47 6EHESIS 21 4,3; 0,9 1.7 0.95 2,8 5,6 226 1.6 2.4 11.1 1,8 2.7
48 HOHARCH 4.9 1,3 2.4 2,20 1.8 3,7 201 1.I 2.5 4.1 1.1 2.6
49 HONARCHPLUS 2 4.9 1.3; 2.4 2.20 l.O 3;.7 201 1.1 2.5 4.1 1.1 2.6
50 EKTACHEH DT 5.2 2.2 3.4 2,13 1.8 2.1 596 1.2 1.3 4.3; 1.0 l.O
51 EKTACHEH 700 3.2 1.6 3;.0 2.66 0.9 1.6 274 1.3; 2.0 5.1 1.2 1.3;
52 PROORE$S 4,9 0.9 1.5 0.96 2.5 4,0 213; 0.9 1,5 5.0 0.6 1.5
53; SPECIFIC N/i)A N/DA N/DA N/DA
54 NOVA 1/1 N/AV N/AV N/AV N/AV
NOVA 4/4 N/AV N/AV N/AV NIAV
, NOVA 5/$ N/AV N/AV N/AV N/AV
57 CODAS FARA 6.5 0.6 1.2 2.50 1.8 2.6 479 1.9 1.4 N/DA
59 CODAS HIRA 6.4 0.9 1.2 2.49 2.2 3.4 400 1.3; 2.0 N/DA
59 ASSIST
60 CHEH
61 RA-IO00
62 RA-2X
RA-500
64 RA-XT
65 9HAC II
t SRA 2000
67 9EH-PROF]LER H/DA
66 ULTROLAD N/DA
69 VITALAD 200 N/DA
N/DA N/DA N/DA NIDA
HIDA NIDA H/DA N/DA
1.6 N/DA 1,9 3.00 H/DA 1.2 150 H/DA 1,5 NIDA
1,0 N/DA 1.9 3,00 N/DA 1,2 150 N/DA 1,5 N/DA
NIDA H/DA H/DA H/DA
NIDA H/DA N/DA N/DA
3,0 1,2 NIDA HIDA 400 2,1 H/DA N/DA
3;,6 1,2 N/DA N/DA 400 2,1 N/DA H/DA
N/DA HIOA H/DA
N/DA NIDA N/DA
N/DA N/DA N/DA
6O
J. Fyffe et al. Review of large biochemistry analysers
Acknowledgements
This work would not have been possible without the
generous help and co-operation of the companies manu-
facturing or marketing the analysers included in this
review.
References and evaluations
Some of the instruments have been subject to DHSS and
other evaluations. The following reports listed by instru-
ment entry number in the data-base are available and can
provide more detailed information.
ESCHLE Code
Abbott VPS
7 Hitachi 705
11 ERIS
28-31 CX3-7
33 Chemilyser
40 Dacos
43 Greiner G400
54 Nova +
61 RA 1000
65 SMAC II
Health Equipment Information
(No. 140, April 1985).
DHSS Evaluation No. STB
3A/85/31.
DHSS Evaluation No. STB
3A/A025.
DHSS Evaluation No. STB
3A/84/25
Callaghan, S. G. et al., Journal
ofAutomatic Chemistry, 7, 90-
94, 1985.
DHSS Evaluation No. A004
1980 and DHSS Evaluation.
Update No. STB 3A/85/32.
DHSS Evaluation No. STB
3A/A021.
DHSS Evaluation No. STB
3A/84/26.
G300 evaluated by Scottish
Home and Health
Department: Health
Equipment Bulletin, 40/83.
DHSS Evaluation No. STB
3A/85/9.
DHSS Evaluation No. STB
3A/A024.
SMAC I DHSS Evaluation
1974.
Reference
FYFFE, J. A., A review of large biochemistry analysers, 1985.
Communications in Laboratory Medicine, I, 118-132.
'CHEMICAL SENSORS CLUB NEWS'
The eighth issue of CSC News (orders to J. A.
Brown, 87 Gower Street, London WC1E 6AA)
includes:
The pocket biosensor arrives; Analyticon: Gas
and vapour sensors; Sensor research at Durham;
Japanese sensor newsletter; Eurosensors 1987;
Club database; Biotechnology Equipment Unit;
School on Process Analysis.
ANALYTICA 88
To be held from 19 to 22 April 1988 in Miinchen,
FR Germany
Analytica is a trade fair combined with an
international conference- the conference this
year being 'Biochemical Analytics'.
About a third of the companies participating in
ANALYTICA- about 600 firms are expected to
register- come from outside FR Germany. It is
anticipated that the largest numbers of exhibi-
tors from abroad will be from the UK (37
companies), the USA (31), Switzerland (18),
France (9) and Italy (10).
In addition to individual exhibitors, France, the
UK, Israel and the USA will also have official
joint presentations. The UK'sjoint presentation
includes 18 exhibitors; the exact numbers of
exhibitors in the presentations of France, Israel
and USA are not yet known.
The Conference
The scientific programme of the th Interna-
tional Conference on Biochemical Analysis, 'Bio-
chemical Analytics 88' (chaired by Professor Dr
Helmut Greiling, Aachen), will again consist of
three parts: symposia, poster exhibition and
ANALYTICA Forum Munich.
In 1986 the conference attracted some 1250
participants. The number ofvisitors is expected
to be far higher in 1988. Some 3200 professionals
from 40 countries have expressed an interest in
the conference.
The symposia will deal with the latest analytical
developments both from the point of view of
methodology and of the various fields ofapplica-
tion. Special attention will be paid to problems of
analytics, such as water analytics, including
environmental biotechnology, as well as modern
methods of molecular biology and gene technol-
ogy.
In addition to the trade fair, the ANALYTICA
Forum Munich provides the exhibiting industry
with the opportunity of giving lectures in which
companies can present and discuss their latest
developments in industrial instrumentation and
methodology.
Forfurther information contact: Miinchener Messe- und
Ausstellungsgesellschaft mbH, Postfach 12 10 09,
D-8000 Munich 12, FR Germany. Tel.: (89) 5107-0,
Telex 5 212 086 ameg d.
61

				

